# PTBP1-Mediated Alternative Splicing Regulates the Inflammatory Secretome and the Pro-tumorigenic Effects of Senescent Cells

**DOI:** 10.1016/j.ccell.2018.06.007

**Published:** 2018-07-09

**Authors:** Athena Georgilis, Sabrina Klotz, Christopher J. Hanley, Nicolas Herranz, Benedikt Weirich, Beatriz Morancho, Ana Carolina Leote, Luana D'Artista, Suchira Gallage, Marco Seehawer, Thomas Carroll, Gopuraja Dharmalingam, Keng Boon Wee, Marco Mellone, Joaquim Pombo, Danijela Heide, Ernesto Guccione, Joaquín Arribas, Nuno L. Barbosa-Morais, Mathias Heikenwalder, Gareth J. Thomas, Lars Zender, Jesús Gil

**Affiliations:** 1 MRC London Institute of Medical Sciences (LMS), Du Cane Road, London W12 0NN, UK; 2 Institute of Clinical Sciences (ICS), Faculty of Medicine, Imperial College London, Du Cane Road, London W12 0NN, UK; 3 Department of Internal Medicine VIII, University Hospital Tübingen, Tübingen 72076, Germany; 4 Department of Physiology I, Institute of Physiology, Eberhard Karls University Tübingen, Tübingen 72076, Germany; 5 Cancer Sciences Unit, Cancer Research UK Centre, University of Southampton, Somers Building, Southampton SO16 6YD, UK; 6 Division of Chronic Inflammation and Cancer, German Cancer Research Centre (DKFZ), Heidelberg 69121, Germany; 7 Preclinical Research Program, Vall d’Hebron Institute of Oncology (VHIO) and CIBERONC, Barcelona 08035, Spain; 8 Instituto de Medicina Molecular, Faculdade de Medicina, Universidade de Lisboa, Lisbon, Portugal; 9 Institute of High Performance Computing, A^∗^STAR, 1 Fusionopolis Way, #16-16 Connexis, Singapore 138632, Singapore; 10 Bioinformatics Institute, A^∗^STAR, 30 Biopolis Street, #07-01 Matrix, Singapore 138671, Singapore; 11 Methyltransferases in Development and Disease Group, Institute of Molecular and Cell Biology, Agency for Science, Technology and Research (A^∗^STAR), Singapore, Singapore; 12 Department of Biochemistry and Molecular Biology, Universitat Autónoma de Barcelona, Campus de la UAB, Bellaterra 08193, Spain; 13 Institució Catalana de Recerca i Estudis Avançats (ICREA), Barcelona 08010, Spain; 14 Translational Gastrointestinal Oncology Group, German Consortium for Translational Cancer Research (DKTK), German Cancer Research Center (DKFZ), Heidelberg 69120, Germany

**Keywords:** SASP, senescence, RNAi screen, alternative splicing, PTBP1, EXOC7, Oncogene-induced senescence

## Abstract

Oncogene-induced senescence is a potent tumor-suppressive response. Paradoxically, senescence also induces an inflammatory secretome that promotes carcinogenesis and age-related pathologies. Consequently, the senescence-associated secretory phenotype (SASP) is a potential therapeutic target. Here, we describe an RNAi screen for SASP regulators. We identified 50 druggable targets whose knockdown suppresses the inflammatory secretome and differentially affects other SASP components. Among the screen candidates was PTBP1. PTBP1 regulates the alternative splicing of genes involved in intracellular trafficking, such as EXOC7, to control the SASP. Inhibition of PTBP1 prevents the pro-tumorigenic effects of the SASP and impairs immune surveillance without increasing the risk of tumorigenesis. In conclusion, our study identifies SASP inhibition as a powerful and safe therapy against inflammation-driven cancer.

## Highlights

• An RNAi screen identifies 50 specific SASP regulators
• The splicing factor PTBP1 regulates a pro-inflammatory SASP subset
• PTBP1 regulates alternative splicing of *EXOC7* to control the SASP
• PTBP1 depletion safely inhibits inflammation-driven cancer

## Graphical Abstract

**Figure:**
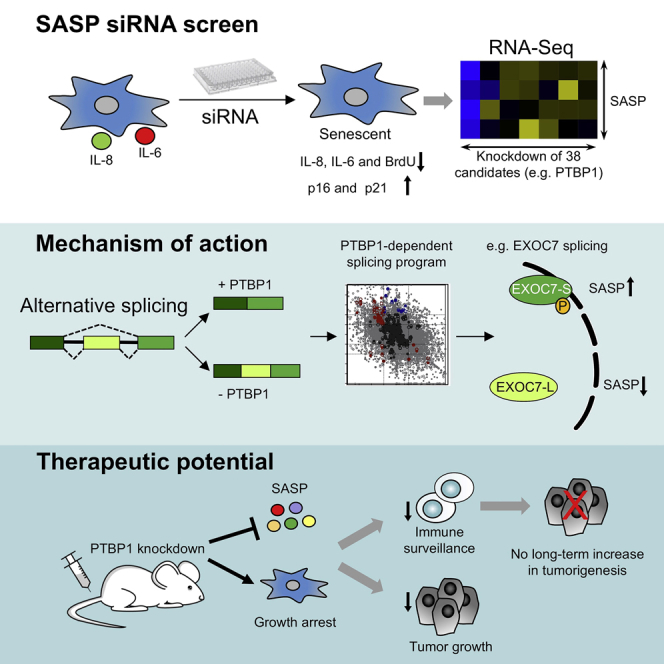

## Teaser

By performing a genetic screen for regulators of the senescence-associated secretory phenotype (SASP), Georgilis et al. identify PTBP1, which controls SASP by regulating alternative splicing of genes involved in intracellular trafficking such as *EXOC7*. PTBP1 knockdown blocks the tumor-promoting functions of SASP.

## Significance

**Oncogene-induced senescence has opposing effects in cancer as it restrains tumor initiation but paradoxically can fuel the growth of advanced tumors via a pro-inflammatory secretome. Here, we identified multiple druggable targets whose inhibition suppresses inflammation without interfering with the senescence growth arrest. One of the identified candidates is PTBP1, a regulator of alternative splicing previously shown to promote cancer proliferation and metastasis. Knockdown of PTBP1 *in vivo* reduced the pro-tumorigenic effects of senescence without increasing the risk of tumor initiation. These findings suggest that targeting the pro-inflammatory SASP is a safe and effective therapeutic strategy that can be employed against inflammation-driven cancers such as advanced liver tumors.**

## Introduction

Senescence is a stress response that limits the replication of damaged or aging cells by implementing a stable growth arrest. Senescent cells display profound changes in nuclear and chromatin organization, gene expression, and cell metabolism (Kuilman et al., 2010). Importantly, senescent cells also secrete a complex combination of mostly pro-inflammatory factors collectively referred to as the senescence-associated secretory phenotype (SASP).

During early tumorigenesis, the SASP adds to the cancer-protective effects of senescence by reinforcing the growth arrest and by signaling to the immune system to clear incipient cancer cells (Acosta et al., 2008, Acosta et al., 2013, Kang et al., 2011). The SASP also contributes to tissue repair and normal development (Munoz-Espin and Serrano, 2014). Conversely, the SASP can mediate many of the detrimental functions of senescent cells. The secretome of lingering senescent cells can promote malignancy of nearby cells (Coppe et al., 2010), chemoresistance (Kaur et al., 2016), and systemic inflammation associated with many age-related diseases (Franceschi and Campisi, 2014).

Although the specific outcome depends on the context, it appears that the net effect of the SASP in advanced cancer is to promote tumorigenesis by enhancing the proliferative and metastatic potential of neoplastic cells, among other mechanisms (Coppe et al., 2010). The harmful inflammation imposed by the SASP suggests that eliminating senescent cells (Ovadya and Krizhanovsky, 2018) or suppressing the SASP can be advantageous in many pathologies and not just cancer.

Several SASP regulators have been identified, most of which drive inflammatory responses. These include nuclear factor κB (NF-κB), CCAAT/enhancer-binding protein β (CEBPβ), p38α MAPK (mitogen-activated protein kinase), mammalian target of rapamycin (mTOR), mixed-lineage leukemia (MLL), GATA4, and Brd4 (Herranz and Gil, 2018). Many of the defined pathways that activate the SASP are by nature important senescence effectors. Consequently, to devise coherent strategies to target the SASP care must be taken not to negate the tumor-suppressive effects associated with the senescence growth arrest. Preliminary evidence indicates that uncoupling cell arrest and the SASP is feasible (Herranz et al., 2015, Laberge et al., 2015, Tasdemir et al., 2016, Wall et al., 2013). Here, we aimed to identify genes that modulate the SASP without interfering with other senescence phenotypes and assess the therapeutic potential of inhibiting the SASP against inflammation-driven cancer.

## Results

### A Small Interfering RNA Screen Identifies SASP Regulators

**Figure 1:**
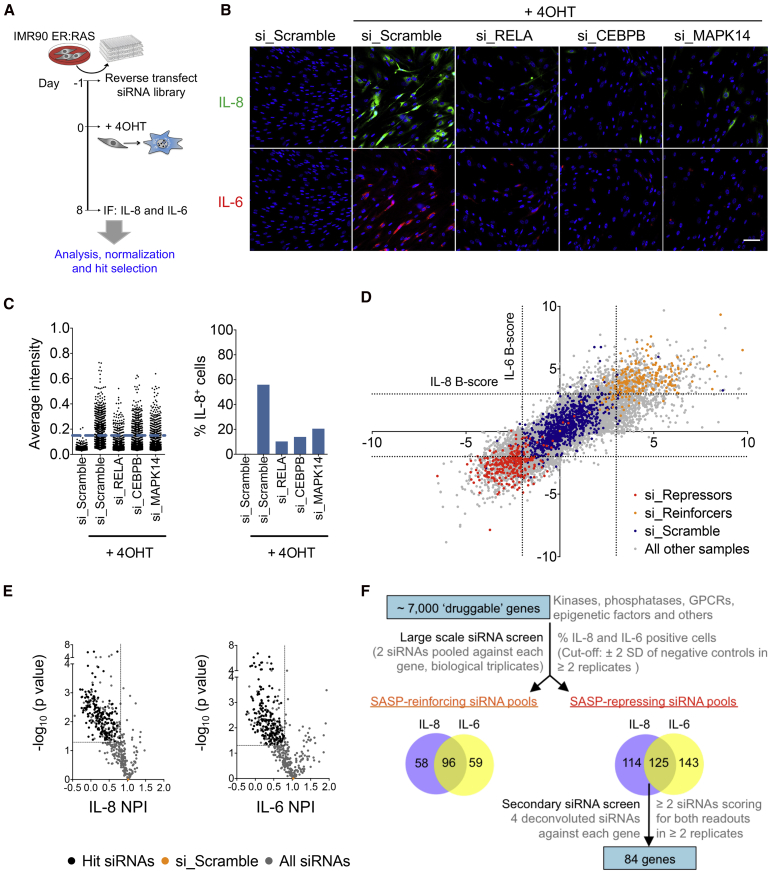
An siRNA Screen Identifies Regulators of the SASP (A) Workflow of the SASP siRNA screen. (B) Representative immunofluorescence (IF) images of IL-8 and IL-6 following transfection of indicated siRNAs. Scale bar, 100 μm. (C) IF quantification. Left panel shows single-cell intensity values of IL-8 in a representative sample well of a 96-well plate seeded with cells transfected with indicated siRNAs. Blue line denotes quantification cutoff resulting in the IL-8 percentages shown in the right panel. (D) Screen results. Normalized IL-8 versus normalized IL-6 values for each replicate sample of the screen. Dotted lines indicate cutoffs of ±2 SD of negative scramble controls. siRNA pools were considered “hits” if they showed a B score of >−2 or <3, in at least 2 out of 3 replicates for both IL-6 and IL-8. (E) Volcano plots of the secondary siRNA screen performed in IMR90 ER:RAS cells as per the workflow given in (A). Normalized percent inhibition (NPI) shown as mean of 3 replicates. Three replicate NPI values of each sample siRNA were compared with all scramble siRNA values by unpaired Student's t test. Eighty-four genes met the selection criteria depicted by lines: ≥2 siRNAs with an IL-8 and IL-6 NPI <0.8 and a p value of ≤0.05. Only siRNAs targeting the 84 genes are color coded as “Hit siRNAs”. (F) Summary of SASP screen. Venn diagrams (not to scale) show number of siRNA pools passing the filter and overlap between IL-8 and IL-6. See also Figure S1.

To discover regulators of the SASP, we carried out a large-scale small interfering RNA (siRNA) screen (Figure 1A). We used IMR90 ER:RAS, a well-characterized cellular system of oncogene-induced senescence (OIS). Activation of RAS with 4-hydroxy-tamoxifen (4OHT) causes IMR90 ER:RAS cells to undergo senescence (Acosta et al., 2013). IMR90 ER:RAS cells treated with 4OHT become growth arrested and express interleukin-8 (IL-8), IL-6, and other SASP components, as analyzed by immunofluorescence (IF) or qRT-PCR (Figures 1B and S1A–S1D). We selected IL-8 and IL-6 as readouts for the screen due to their significant induction during OIS and the relevance of these cytokines in mediating SASP-related phenotypes (Acosta et al., 2008, Kuilman et al., 2008). After monitoring the kinetics of IL-8 and IL-6 expression during OIS (Figures S1C and S1D), we decided to carry out the screen 8 days after 4OHT induction. Importantly, transfection of siRNAs targeting known SASP regulators such as the RELA subunit of NF-κB, CEBPβ, or MAPK14, which encodes for p38α, decreased IL-8 and IL-6, as quantified using an automated high-throughput microscopy system (Figures 1B, 1C, and S1E). We screened a “druggable genome” siRNA library targeting around 7,000 genes and identified 96 genes whose knockdown increased IL-8 and IL-6, and 125 genes whose knockdown downregulated IL-8 and IL-6 during OIS (Figure 1D). We validated the siRNAs repressing the SASP in a secondary screen using a new library containing four siRNAs targeting each of the aforementioned 125 candidates (Figure 1E). At least two independent siRNAs prevented the induction of IL-8 and IL-6 during OIS for 84 of the 125 candidates tested (Figures 1E and 1F).

### Identifying SASP Regulators that Do Not Revert the Senescence Growth Arrest

The siRNA screen described above identified 84 potential SASP regulators. However, it was unclear whether knocking down these genes reduced IL-8 and IL-6 by preventing SASP induction specifically or by preventing senescence. Although genes belonging to either group are of biological interest, strategies to repress the SASP while maintaining the senescence growth arrest are appealing to target inflammation-driven tumorigenesis (Coppe et al., 2010, Tchkonia et al., 2013).

**Figure 2:**
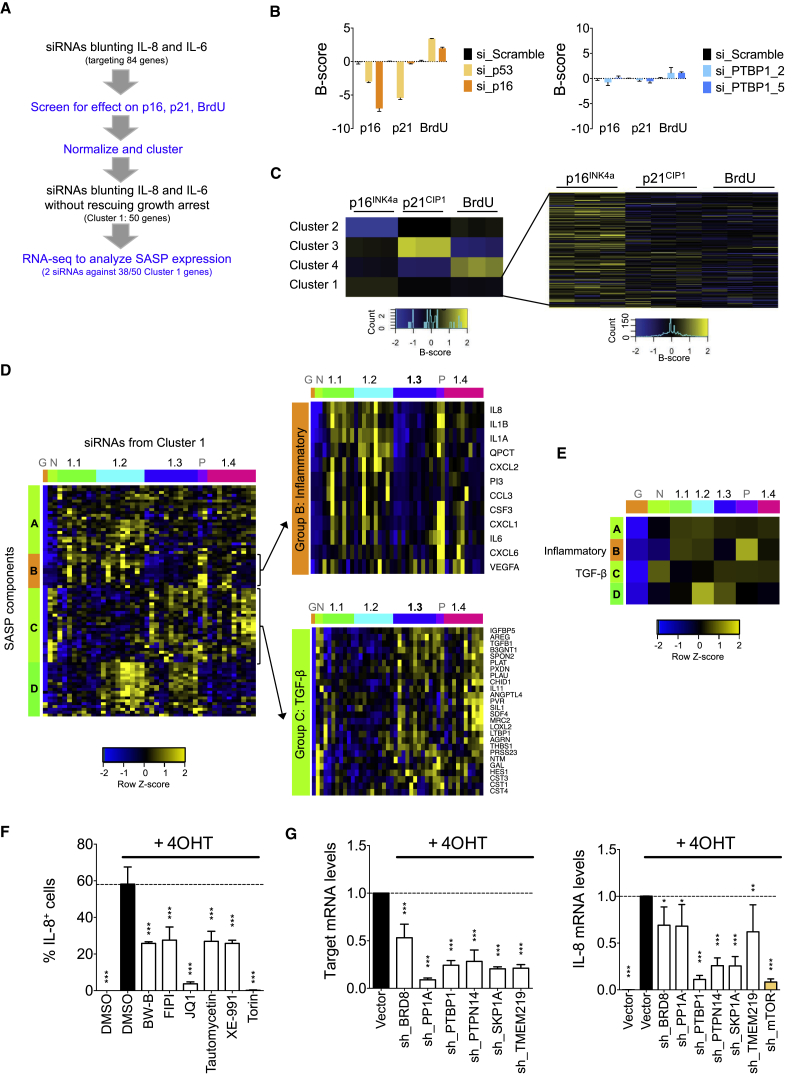
A Subset of Screen Candidates Differentially Regulates the SASP without Affecting the Senescent Growth Arrest (A) Workflow for the categorization of SASP-repressing siRNAs. (B) B scores showing the effects of different siRNAs on the expression of p16 and p21 and incorporation of BrdU. Left: positive controls. Right: two independent siRNAs targeting a gene representative of cluster 1. Data represent mean ± SD (n = 3). (C) *K*-means clustering of the SASP-repressing siRNAs. Heatmap of cluster 1 showing B-score expression for each replicate experiment (column). Each row reflects the measures from one siRNA. (D) Heatmap showing the differential regulation of SASP components by siRNAs targeting cluster 1 candidates. IMR90 ER:RAS cells were independently transfected with two siRNAs targeting 38 of the cluster 1 candidates. RNA-seq was performed and samples clustered according to the expression of SASP components. G: growing cells including cells transfected with scramble siRNA but not treated with 4OHT (no SASP induction), N: grouping senescent cells transfected with siRNAs targeting CEBPβ and RELA (preventing the induction of many SASP components), Pos: cluster grouping senescent cells transfected with scramble siRNA or a siRNA targeting p16 (showing a “maximum” SASP induction). Each column represents an average of two siRNAs per gene with three replicates each. Left: hierarchical cluster showing the siRNA subclusters (colored horizontally) and the clustering of SASP components in groups (colored vertically). Right: zoom-in showing the effect of siRNAs on indicated SASP groups. (E) Summary heatmap derived by averaging the heatmap presented in (D) showing differential regulation of SASP components. (F) IL-8 IF analysis 8 days after senescence induction of IMR90 ER:RAS cells treated with indicated drugs targeting cluster 1 genes. Torin 1 was included as control. Data represent mean ± SD (n = 3); ^∗∗∗^p < 0.001. Comparisons with DMSO + 4OHT. (G) Expression levels of each gene or IL-8 measured by qRT-PCR 6 days after 4OHT induction of IMR90 ER:RAS cells stably infected with an empty pGIPZ vector (Vector) or pools of four pGIPZ-based shRNAs against the indicated candidate SASP regulators. An shRNA targeting mTOR (sh_mTOR) was included as control. Data represent mean ± SD (n = 3); ^∗^p < 0.05, ^∗∗^p < 0.01, ^∗∗∗^p < 0.001. Comparisons with Vector + 4OHT. One-way ANOVA (Bonferroni’s test) was used in (F) and (G) to calculate statistical significance. See also Figure S2 and Table S1.

To identify genes that regulate the SASP without reverting the senescence growth arrest, we assessed how the candidate siRNAs affected OIS-mediated induction of the senescence effectors p16^INK4a^ and p21^CIP1^ and of growth arrest, monitored by IF and assessing bromodeoxyuridine (BrdU) incorporation, respectively (Figure 2A). As controls, we transfected siRNAs targeting either p16^INK4a^ or p53 and confirmed that these siRNAs caused downregulation of p16^INK4a^ or p21^CIP1^, respectively, and blunted the senescence growth arrest as assessed by increased BrdU incorporation (Figures 2B [left panel] and S2A). By using *K*-means clustering, we classified the SASP-repressing siRNAs in four categories (Figures 2C and S2B). Clusters 2 and 4 encompassed siRNAs that prevented the senescence growth arrest as well as induction of p16^INK4a^ or p21^CIP1^, whereas those in cluster 3 further upregulated p21^CIP1^ exacerbating the arrest (Figures S2B and S2C). Importantly, cluster 1 contained siRNAs that reduced IL-8 and IL-6 without reverting the senescence response, satisfying our criteria for genes that are required for SASP but not growth arrest (Figures 2B [right panel] and 2C). Fifty genes grouped in this cluster (Table S1).

Although we used IL-8 and IL-6 as readouts of our screen, the SASP comprises dozens if not hundreds of secreted factors (Acosta et al., 2013, Coppe et al., 2010). To investigate how the identified candidates regulate the SASP, we performed genome-wide transcriptome profiling. We targeted 38 out of the 50 genes found in cluster 1 with two siRNAs against each. To organize the transcriptome data, we used a gene set consisting of the SASP components induced in IMR90 cells undergoing OIS (Acosta et al., 2013, Herranz et al., 2015). The analysis categorized the candidate genes from cluster 1 into four different subclusters (1.1, 1.2, 1.3, and 1.4) demonstrating differential SASP regulation (Figures 2D, 2E, and S2D). All the candidates analyzed prevented IL-8 and IL-6 induction and the expression of most inflammatory SASP components clustering in group B (in Figure 2E, compare row B with maximum SASP induction, “P”). However, differential effects were observed on the expression of other SASP components such as transforming growth factor β and pro-fibrotic SASP components (clustering in group C). The summary heatmap (Figure 2E) also reveals that the siRNAs grouped in subclusters 1.3 and 1.4 were more potent inhibitors of the inflammatory subset of the SASP (see also Figure 2D, top right).

To validate our candidates and verify their therapeutic potential, we utilized chemical inhibitors targeting five candidates from cluster 1 or close family members: BW-B (inhibiting ALOX5), FIPI (inhibiting phospholipase D), JQ1 (inhibiting BET family proteins, related to BRD8), Tautomycetin (inhibiting protein phosphatase 1), XE-991 (inhibiting Kv7 voltage-gated potassium channels), and Torin (mTOR, positive control) (Herranz et al., 2015). Treatment with these inhibitors blunted the induction of IL-8 and IL-6 without rescuing the senescence growth arrest (Figures 2F and S2E). Similarly, pools of four short hairpin RNAs (shRNAs) targeting six cluster 1 genes (*BRD8*, *PP1A*, *PTBP1*, *PTPN14*, *SKP1A*, and *TMEM219*; Figure 2G, left panel) attenuated IL-8 and IL-6 induction (Figure 2G, right panel and Figure S2F, left panel) without reverting the growth arrest (Figures S2E and S2F, right panels). Thus, chemical inhibitors and shRNAs phenocopied the corresponding siRNAs, confirming that our screen successfully identified regulators that differentially affect SASP composition without disturbing the senescence growth arrest.

### PTBP1 Regulates the SASP without Affecting Other Senescence Phenotypes

Among the SASP regulators identified in the screen, knocking down the polypyrimidine tract binding protein 1 (PTBP1) had a strong effect on the pro-inflammatory SASP. PTBP1 encodes for a regulator of alternative splicing whose expression positively correlates with tumor growth and poor prognosis (Wang et al., 2017, Xue et al., 2009).

**Figure 3:**
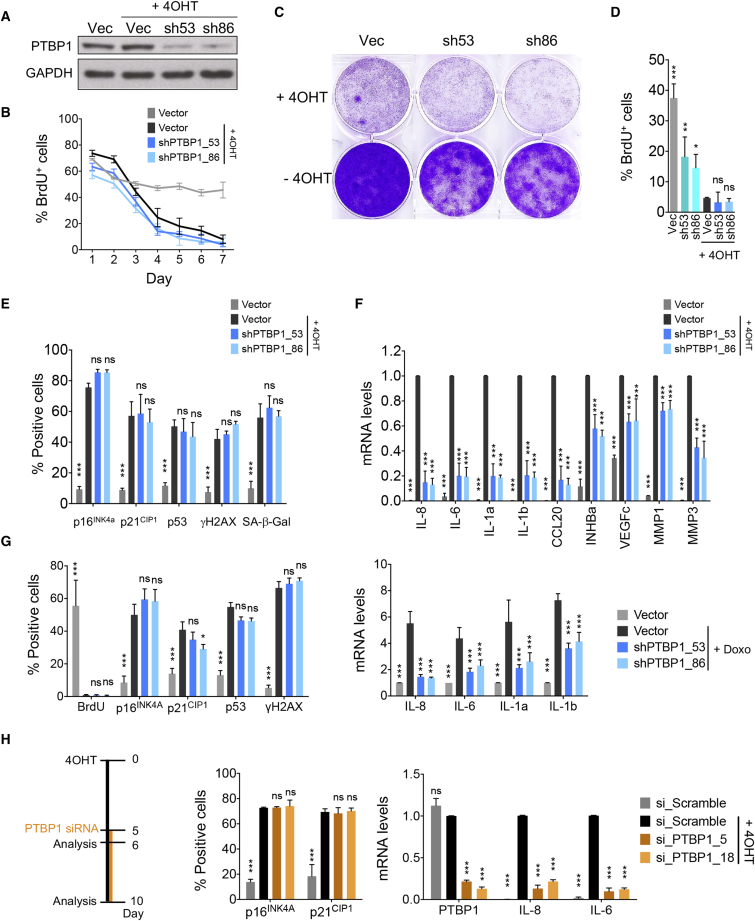
The Splicing Factor PTBP1 Regulates the SASP without Affecting Growth Arrest (A) Immunoblot of protein extracts 6 days after 4OHT induction of IMR90 ER:RAS cells infected with indicated pGIPz shRNA vectors targeting PTBP1. Vec, empty vector. (B) Quantification of cells positive for BrdU incorporation at indicated days after 4OHT treatment. Data represent mean ± SD (n = 3). (C) Crystal violet-stained 6-well dishes of cells fixed 12 days following 4OHT treatment. (D) Quantification of BrdU incorporation 8 days after 4OHT treatment, 15 days after empty vector or PTBP1 shRNA infection. Data represent mean ± SD (n = 3); ^∗^p < 0.05, ^∗∗^p < 0.01, ^∗∗∗^p < 0.001; ns, not significant. Comparisons with Vec + 4OHT. One-way ANOVA (Dunnett's test). (E) Quantification of cells positive for the senescence markers p16, p21, p53, and γH2AX 6 days after 4OHT and β-galactosidase 8 days after 4OHT by IF analysis. Data represent mean ± SD (n = 3). ^∗∗∗^p < 0.001; ns, not significant. Comparisons with Vector + 4OHT, two-way ANOVA (Bonferroni’s test). (F) Expression levels of the indicated SASP genes assessed by qRT-PCR 6 days after 4OHT induction normalized and compared with Vector + 4OHT. Data represent mean ± SD (n = 3); ^∗∗∗^p < 0.001, two-way ANOVA (Dunnett's test). (G) IMR90 WT cells were infected with indicated pGIPZ empty vector or PTBP1 shRNAs and treated with doxorubicin to induce senescence. Left: IF analysis of the indicated senescence markers 6 days after doxorubicin induction. Right: mRNA analysis of the indicated genes by qRT-PCR (right) 8 days after doxorubicin induction normalized to the Vector + doxycycline (Doxo) condition. Data represent mean ± SD (n = 3). ^∗^p < 0.05, ^∗∗∗^p < 0.001; ns, not significant. Comparisons with Vector + Doxo, two-way ANOVA (Dunnett's test). (H) IMR90 ER:RAS cells were transfected with two independent siRNAs targeting PTBP1 at day 5 after senescence induction as indicated in the scheme (left). Senescence establishment at day 6 was monitored by IF analysis (middle). Knockdown of PTBP1 and the effect on the indicated genes was assessed by qRT-PCR 5 days after siRNA transfection, and 10 days after senescence induction (right), normalized to the si_Scramble + 4OHT condition. Data represent mean ± SD (n = 3). ^∗∗∗^p < 0.001; ns, not significant. Comparisons with si_Scramble + 4OHT, two-way ANOVA (Dunnett's test). See also Figure S3.

To study the function of PTBP1 during OIS, we used two independent shRNAs targeting human PTBP1. Both shRNAs efficiently knocked down PTBP1 expression (Figures 3A and S3A). In agreement with our data above, PTBP1 knockdown did not prevent the growth arrest observed during OIS (Figures 3B and 3C). In fact, knockdown of PTBP1 resulted in slightly slower proliferation of normal cells (Figures 3C and 3D). A similar induction of senescence markers, including SA-β-galactosidase activity, the DNA damage response, and upregulation of p16^INK4a^, p21^CIP1^, and p53 expression was observed in senescent cells irrespective of PTBP1 depletion (Figures 3E and S3B). In contrast, PTBP1 knockdown prevented the induction of a specific subset of SASP components that included inflammatory factors such as IL-8, IL-6, and IL-1α (Figure 3F).

The ability of PTBP1 depletion to dampen the SASP without affecting other parts of the senescence program was not restricted to RAS-induced senescence nor was it unique to IMR90 cells. Knockdown of PTBP1 prevented SASP induction in response to doxorubicin in IMR90 cells (Figures 3G and S3C), irradiation in human fibroblasts (Figure S3D), and oncogenic HER2 expression in MCF7 breast cancer cells (Figure S3E), without reverting the growth arrest (Figures 3G, S3D, and S3E). Moreover, PTBP1 depletion also prevented RAS-dependent induction of IL-8 and IL-6 in cells that were incapable of undergoing senescence due to p53 depletion (Figure S3F), suggesting that PTBP1 could control inflammation in settings other than senescence. Finally, knocking down PTBP1 in already senescent cells also decreased IL-8 and IL-6 levels (Figure 3H). Therefore, PTBP1 reduction not only prevents SASP induction but can also inhibit the SASP once senescence has been established. Overall, these results reveal that depletion of PTBP1 represses the SASP without affecting other features of senescence.

### PTBP1 Regulates a Pro-inflammatory SASP Subset Affecting Its Paracrine Functions

**Figure 4:**
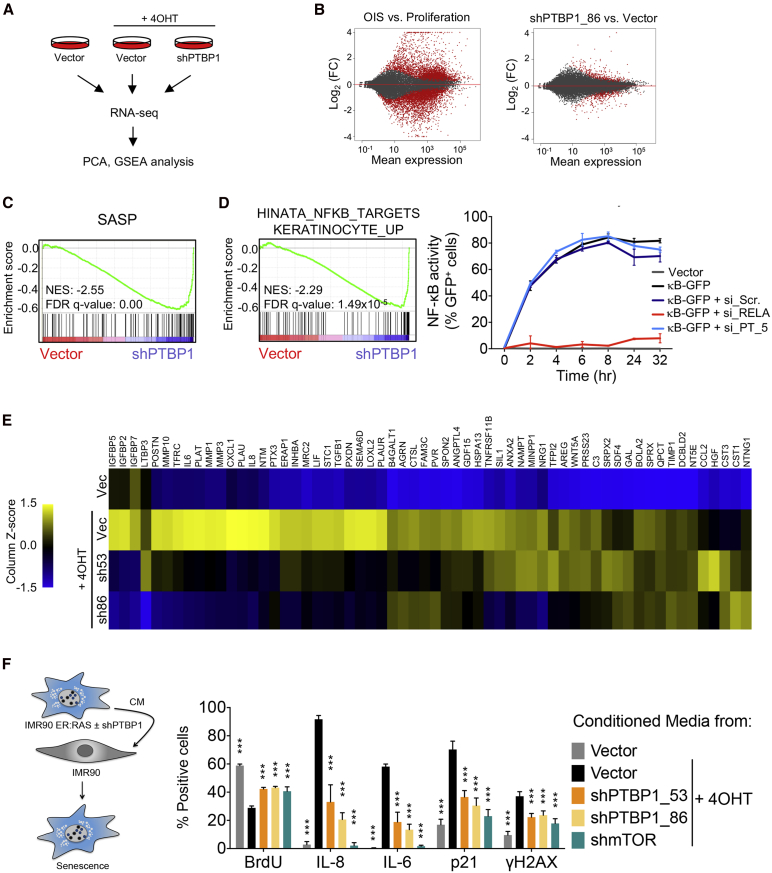
PTBP1 Regulates a Pro-inflammatory Subset of the SASP and Its Paracrine Functions (A) Experimental design for the global transcriptional profiling of IMR90 ER:RAS cells (6 days after 4OHT induction) presented in (B) to (D). (B) Subset of senescence-specific transcripts affected by PTBP1 knockdown. Mean expression (average of the normalized read counts for 3 replicates) in relation to log_2_ (FC) for the indicated comparison. Significantly changing genes are highlighted in red. (C) SASP GSEA signature in PTBP1 depleted cells + 4OHT compared with cells expressing empty vector + 4OHT. (D) NF-κB GSEA signatures in PTBP1 depleted cells + 4OHT compared with cells expressing empty vector + 4OHT (left). GFP analysis at indicated time points following TNFα (50 ng/mL) treatment in IMR90 cells expressing a κB reporter (κB-GFP) and transfected with indicated siRNAs (right). Data represent mean ± SD (n = 3). (E) Mass spectrometry analysis of CM collected from IMR90 ER:RAS cells (empty vector or two PTBP1-targeting shRNAs) 6 days after senescence induction with 4OHT. Differential secretion of the listed SASP factors shown as mean (n = 3). (F) Experimental design to assess the effect of PTBP1 loss on secreted factors responsible for inducing paracrine senescence (left). IF analysis of the senescence markers in IMR90 cells treated for 3–4 days with CM from the indicated IMR90 ER:RAS cells. Data represent mean ± SD (n = 3); each replicate experiment corresponds to independent generation of CM. ^∗∗∗^p < 0.001. Comparisons with cells treated with CM from Vector + 4OHT, two-way ANOVA (Dunnett's test). See also Figure S4.

To better understand the extent of PTBP1-mediated SASP regulation, we analyzed the transcriptome of senescent cells upon PTBP1 knockdown (Figure 4A). While senescence induction resulted in substantial changes in gene expression, only a small set of these changed upon PTBP1 knockdown (Figure 4B). Principal component analysis (PCA) substantiated this observation (Figure S4A), and gene set enrichment analysis (GSEA) further confirmed that depletion of PTBP1 inhibited the SASP without interfering with the growth arrest (Figures 4C and S4B). Analysis of the transcriptome data found NF-κB-dependent signatures were downregulated upon PTBP1 knockdown (Figures 4D [left panel] and S4B). We investigated whether that reflected direct regulation of NF-κB signaling by PTBP1 or was the result of reduced SASP disrupting the positive feedback loop signaling needed to amplify SASP expression. To this end, IMR90 cells bearing an NF-κB reporter (Natarajan et al., 2014) were treated with tumor necrosis factor α (TNFα). While knocking down RELA prevented NF-κB activation, knocking down PTBP1 did not affect it (Figure 4D). These experiments suggest that the inhibition of NF-κB signaling observed upon PTBP1 knockdown is caused by the reduced SASP impairing the NF-κB-dependent SASP autocrine loop.

Next, we cataloged the factors secreted by senescent cells using label-free mass spectrometry (Acosta et al., 2013, Herranz et al., 2015). We identified 60 SASP components enriched in the conditioned media (CM) of senescent cells, and the knockdown of PTBP1 reduced the levels of 32 (Figure 4E), including many pro-inflammatory cytokines. We next explored how PTBP1 depletion affected different paracrine actions mediated by senescent cells. First, we examined whether PTBP1 depletion could affect paracrine senescence (Acosta et al., 2013). Naive IMR90 cells treated with CM from senescent cells undergo senescence and express an SASP (Acosta et al., 2013). These effects were impaired in naive cells treated with CM from PTBP1-depleted senescent cells (Figure 4F). Conversely, treatment with senescent CM induced paracrine arrest on cells depleted of PTBP1 but resulted in a reduced secondary SASP (Figure S4C).

### PTBP1 Knockdown Inhibits the Tumor-Promoting Functions of the SASP

**Figure 5:**
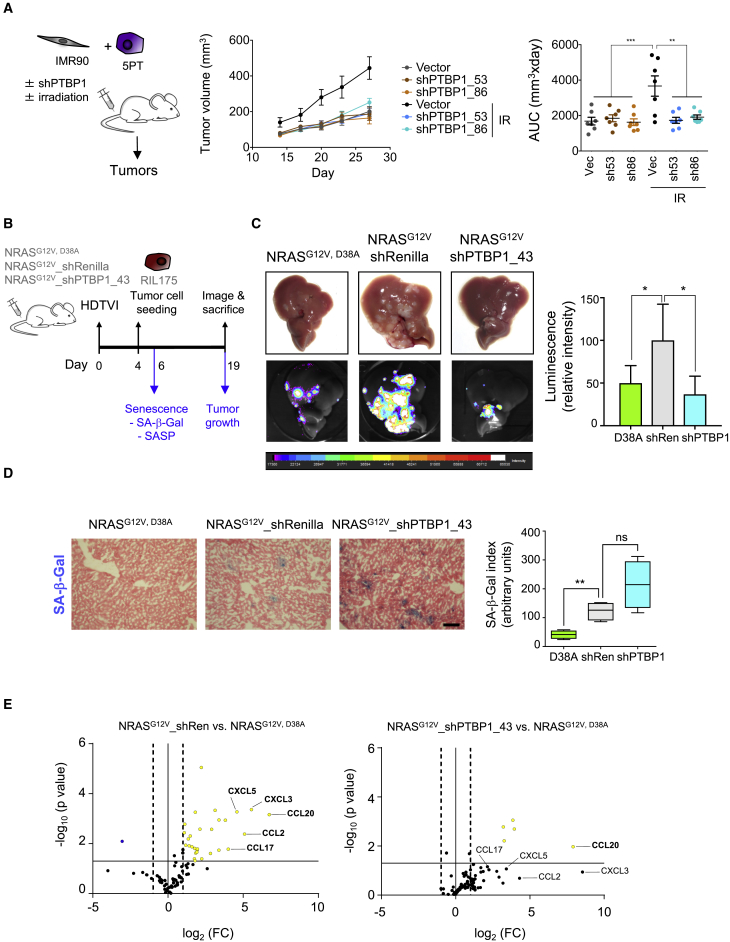
PTBP1 Knockdown Inhibits the Tumor-Promoting Functions of the SASP (A) Tumor growth induced by senescent cells in a xenograft mouse model following PTBP1 knockdown. Left: experimental design. Middle: tumor growth monitored by measuring the volume at the indicated days. Graph symbols are mean volumes of all the mice in the indicated condition. Right: area under the curve (AUC) of the tumor growth for each mice. Data represent mean ± SD (n = 7 per group). ^∗∗^p < 0.01, ^∗∗∗^p < 0.001, one-way ANOVA (Bonferroni’s test). (B–E) Tumor growth in an orthotopic model of advanced liver cancer following PTBP1 knockdown. (B) Experimental design. (C) Representative images of livers and luciferase imaging (left) and quantification of luciferase intensity (right) shown as mean ± SD (n = 4 mice per group). ^∗^p < 0.05, one-way ANOVA (Bonferroni’s test). (D) Representative images and quantification of SA-β-Galactosidase expression. Scale bar: 50 μm. Plots show median (line), upper and lower quartiles (boxes), and lines extending to highest and lowest observation (whiskers), ^∗∗^p < 0.01; ns, not significant; one-way ANOVA (Bonferroni’s test). (E) qRT-PCR-based quantification of SASP components shown as log_2_ (FC) between the conditions indicated at the top. See also Figure S5.

The SASP can enhance the proliferative potential of cancer cells to promote tumor progression (Coppe et al., 2010). To investigate how PTBP1 depletion affects the tumor-promoting functions of the SASP, we used an experimental xenograft mouse model that monitors the effect of senescent fibroblasts on tumor growth (Herranz et al., 2015). We co-injected squamous cell carcinoma 5PT cells with normal or senescent (irradiated) fibroblasts subcutaneously into nude mice and confirmed that the presence of senescent fibroblasts enhanced tumor growth (Figure 5A). Depletion of PTBP1 impaired the ability of irradiated fibroblasts to promote the growth of 5PT tumor cells in this setting (Figures 5A, S5A, and S5B). These experiments suggest that knocking down PTBP1 suppresses the ability of senescent fibroblasts to promote tumor growth. However, the use of an immunocompromised mouse model does not fully capture the complex interactions occurring in the tumor microenvironment.

Senescent hepatocytes are present in damaged livers (Jurk et al., 2014, Krizhanovsky et al., 2008), and during chronic liver disease precancerous senescent hepatocytes co-exist with readily transformed cells. A mouse model recapitulating this interaction was recently published (Eggert et al., 2016) (Figure 5B). In this model, senescence is induced in wild-type (WT) mouse livers by transposon-mediated transfer of oncogenic NRAS (NRAS^G12V^) by hydrodynamic tail vein injection. Transduction of a non-oncogenic NRAS (NRAS^G12V, D38A^) serves as a control (Kang et al., 2011). After 4 days, hepatocellular carcinoma cells expressing firefly luciferase are seeded in the liver of syngeneic mice, and tumor growth can be monitored by *ex vivo* tumor imaging (Figure 5B). Importantly, this model allows us to assess the growth of orthotopic liver tumors in fully immunocompetent mice, hence in a context more relevant to human disease.

To study the therapeutic value of targeting PTBP1, we expressed NRAS^G12V, D38A^, an inactive NRAS mutant (NRAS^G12V, D38A^) that does not induce senescence (Kang et al., 2011), and co-expressed NRAS^G12V^ with either a neutral shRNA (shRen, targeting *Renilla* luciferase) or shRNAs targeting PTBP1 in the same vector (Figure S5C). To assess the effect that depletion of PTBP1 in senescent hepatocytes has on tumor growth, we seeded the syngeneic tumor cells, and after 15 days evaluated the tumors macroscopically and by luciferase imaging. Knockdown of PTBP1 in senescent NRAS^G12V^-expressing hepatocytes prevented the acceleration of tumor growth otherwise observed in senescent livers (NRAS^G12V^_shRen) compared with non-senescent livers (NRAS^G12V, D38A^) (Figure 5C). In accordance with what we observed in cell culture, depleting PTBP1 expression did not inhibit senescence in the liver (Figure 5D) but significantly reduced the expression of multiple SASP components (Figure 5E). In summary, these data provide functional proof in a preclinical disease model of the feasibility of SASP modulation as a strategy to attenuate tumor growth.

### PTBP1 Knockdown Affects Senescence Surveillance but Does Not Increase the Risk of Tumorigenesis

**Figure 6:**
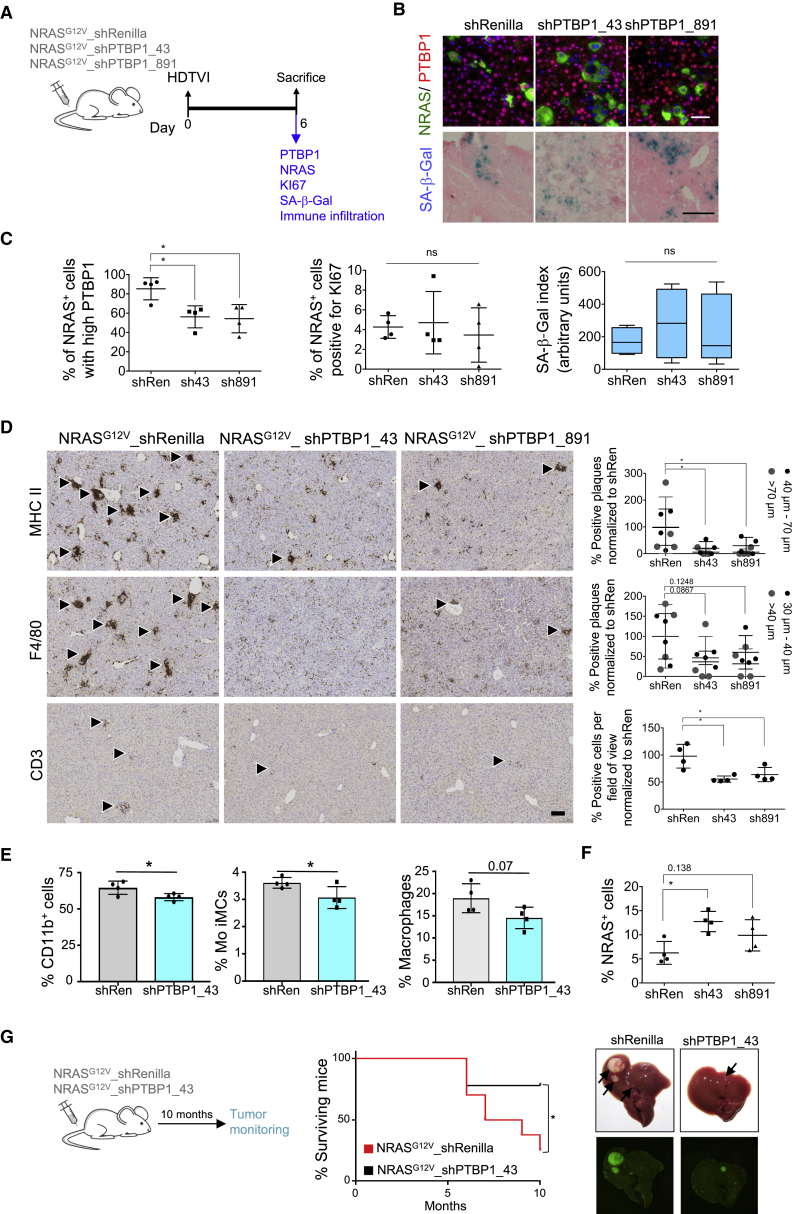
PTBP1 Knockdown Impairs Senescence Surveillance without Increasing Tumorigenesis (A–F) Senescence surveillance following PTBP1 knockdown. (A) Experimental design. (B) Representative IF images of NRAS, PTBP1 and SA-β-galactosidase expression in livers. Scale bars, 50 μm. (C) Quantification of high PTBP1-expressing or Ki67^+^ among NRAS^+^ cells by IF. Quantification of SA-β-gal expression is also shown (right). Plots show median (line), upper and lower quartiles (boxes), and lines extending to highest and lowest observation (whiskers). Data represent mean ± SD (n = 4). ^∗^p < 0.05; ns, not significant. Comparisons with NRAS^G12V^-shRenilla, one-way ANOVA (Dunnett's test). (D) Representative IHC images (left) and densitometric quantification (right) of indicated immune cell markers. For MHCII^+^ and F4/80^+^ areas, arrowheads indicate characteristic myeloid aggregate formation that develops as a consequence of NRAS^G12V^-driven senescence in the liver. Smaller aggregates are separated from larger aggregates based on diameter (comparison shown in Figures S6A and S6B) and are depicted as black and gray symbols, respectively. For CD3^+^ staining, arrowheads indicate positive cells and are quantified as number of positive cells per counting area (10 mm^2^). Scale bar, 100 μm. Data represent mean ± SD (n = 4). ^∗^p < 0.05. Comparisons with NRAS^G12V^-shRenilla, one-way ANOVA (Bonferroni’s test). (E) Quantification of indicated infiltrating immune cells by flow cytometry. Gating strategy shown in Figures S6C and S6D. Data represent mean ± SD (n = 4); ^∗^p < 0.05. Comparisons with NRAS^G12V^-shRenilla, one-way ANOVA (Bonferroni’s test). (F) Quantification of NRAS^+^ cells. Data represent mean ± SD (n = 4); ^∗^p < 0.05. Comparisons with NRAS^G12V^-shRenilla, one-way ANOVA (Bonferroni’s test). (G) Long-term tumorigenesis in WT mice upon injection with indicated transposon-based plasmids. Left: experimental design. Middle: Kaplan-Meier survival curves. Right: NRAS^G12V^_shRen (n = 10) and NRAS^G12V^_shPTBP1 (n = 9). ^∗^p < 0.05 by log-rank (Mantel-Cox) test. Representative images of macroscopically visible GFP^+^ tumor nodules (>1 mm, black arrows) at endpoint. See also Figure S6.

While the SASP is thought to mediate many of the detrimental effects attributed to senescent cells during aging and cancer, it also has protective functions. Notably, factors secreted by senescent cells are necessary to mount a protective immune surveillance response during tumor initiation or in response to re-engagement of senescence in tumors (Kang et al., 2011). Therefore, we decided to evaluate the effect of PTBP1 depletion on the surveillance and elimination of incipient preneoplastic hepatocytes (Figure 6A). Mice injected with NRAS^G12V^_shPTBP1 showed lower PTBP1 expression in NRAS^+^ hepatocytes compared with mice injected with NRAS^G12V^_shRen (Figures 6B and 6C). PTBP1 depletion did not significantly alter the percentage of proliferating NRAS^+^ cells (Ki67 staining) and the SA-β-galactosidase activity was not significantly different between NRAS^G12V^_shPTBP1 and NRAS^G12V^_shRen mice (Figures 6B and 6C).

Since PTBP1 knockdown in senescent hepatocytes results in reduced SASP production (Figure 5E), we investigated how this affects immune cell recruitment. We first carried out immunohistochemistry (IHC) staining and found that the formation of macrophage aggregates (F4/80 and MHC II staining) and the infiltration of T cells (CD3 staining) was significantly reduced in the livers of NRAS^G12V^_shPTBP1 (Figures 6D, S6A, and S6B). Next, we measured CD11b^+^ infiltrating immune cells using flow-cytometry analysis (Figures S6C and S6D). This is highly relevant since different populations of myeloid cells have been implicated in both senescent immune surveillance (Kang et al., 2011) and mediating the tumor-promoting effects of senescent cells in damaged livers (Eggert et al., 2016). Consistent with previous observations (Eggert et al., 2016, Kang et al., 2011), expression of oncogenic NRAS (NRAS^G12V^) resulted in significant infiltration of CD11b^+^ cells and monocyte immature myeloid cells (Mo iMC), and a slight (not statistically significant) increase in macrophage infiltration (Figure S6E). Depletion of PTBP1 decreased the infiltration of all these immune cells, although it was not statistically significant (Figure 6E). Macrophages can be further subdivided in Kupffer cells and newly infiltrating macrophages (Figure S6C). Although both subpopulations increased in NRAS^G12V^-injected livers, only the recruitment of new macrophages was reduced by PTBP1 knockdown, although not significantly (Figures S6F and S6G). A corollary of the impaired immune infiltration upon PTBP1 knockdown would be impaired senescent cell clearance. A reduced percentage of NRAS^+^ hepatocytes 6 days after transduction was observed when comparing NRAS^G12V^ and the senescent incapable NRAS^G12V, D38A^ control (Figure S6H). This reduction was impaired upon PTBP1 knockdown, as noted by an increase in the percentage of NRAS^+^ cells in those mice (Figure 6F).

Next, we evaluated whether the impaired immune surveillance caused by PTBP1 knockdown resulted in increased long-term risk in tumorigenesis. To this end, mice were transduced with vectors co-expressing NRAS^G12V^ and an shRNA targeting PTBP1 (shPTBP1_43) or a neutral control shRNA (shRen; Figure 6G, left panel). Tumor formation was monitored periodically by sonography (Figure S6I). Interestingly, knockdown of PTBP1 did not impart an increased risk of tumor formation, but rather resulted in increased survival and decreased tumor formation (Figures 6G and S6J). Overall, these data suggest that therapeutic SASP modulation to treat advanced cancers is not only effective and feasible but can also be safe, without running the risk of tumor initiation due to bypass of lingering senescent cells or dampened anti-tumor immunity.

### Regulation of Alternative Splicing by PTBP1 Controls the SASP

**Figure 7:**
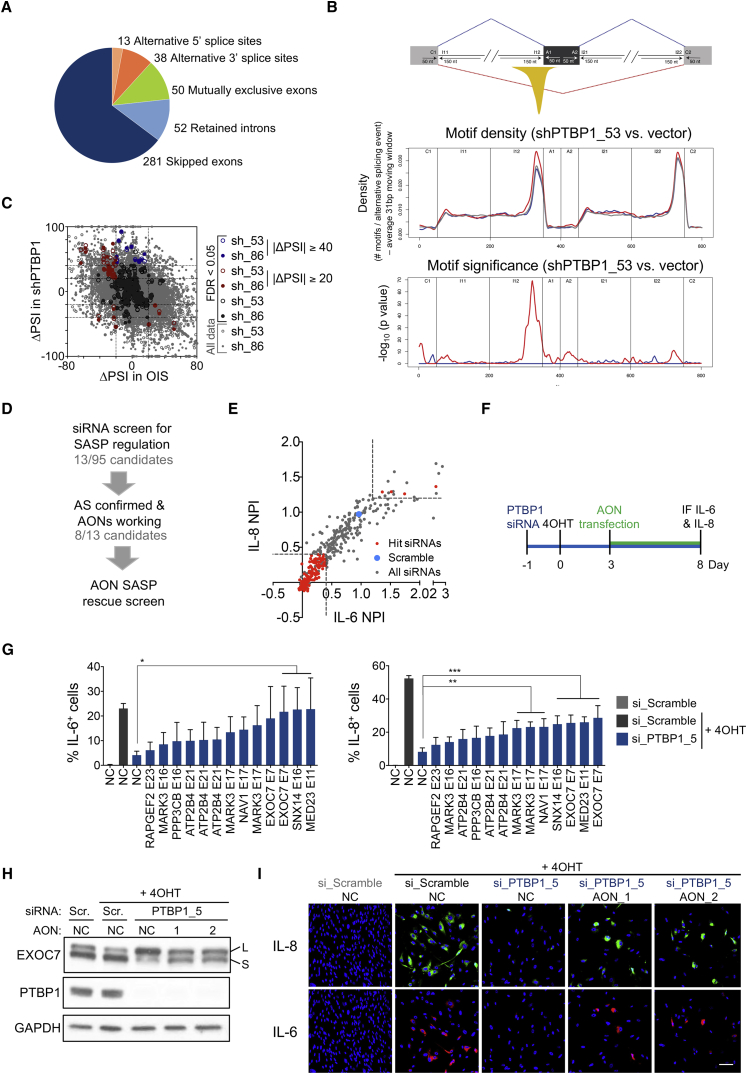
Regulation of Alternative Splicing by PTBP1 Controls the SASP (A) Distribution of the five types of AS events detected in senescent cells compared with proliferating cells by RNA-seq (see Figure 4A). (B) PTBP1 RNA binding motifs across alternative exons upon PTBP1 knockdown. Top: scheme. Motifs are mapped to potential regulatory sequences around the target alternatively spliced exon (dark-gray box). The yellow peak represents the area of predicted enrichment of PTBP1 binding responsible for exon splicing repression (red line), with no role known for PTBP1 in exon splicing enhancement (dashed blue line). Middle: motif density for exons with inclusion increasing (putatively repressed, red), decreasing (putatively enhanced, blue), or not altered (not regulated, gray) upon PTBP1 knockdown. Bottom: statistical significance for local motif enrichment in putatively repressed (red) and enhanced (blue) exons. (C) Exon-skipping events and ΔPSI cutoffs used for shortlisting events changing due to loss of PTBP1. A stricter cutoff was used for events changing upon PTBP1 loss but not affected upon senescence. (D) Strategy to link PTBP1-driven alternative splicing and SASP regulation. (E) Ninety-five PTBP1-spliced genes were targeted with four siRNAs and screened for IL-8 and IL-6 regulators as described in Figure 1. NPI shown as mean of three replicates and cutoffs for hit selection (dotted lines). Hit siRNAs represent siRNAs targeting genes scoring with ≥2 siRNAs in both readouts. (F) Experimental design of (G). (G) IMR90 ER:RAS cells were transfected with AONs either not targeting (NC) or targeting the indicated exons. IF analysis of IL-6 (left) and IL-8 (right). Data represent mean ± SD (n = 4). ^∗^p < 0.05, ^∗∗^p < 0.01, ^∗∗∗^p < 0.001. Comparisons with NC, si_PTBP1_5 + 4OHT, one-way ANOVA (Dunnett's test). (H and I) Effect of AONs targeting EXOC7 exon 7 splicing on the SASP downregulation caused by PTBP1 knockdown. Timeline as in (F). (H) Immunoblot of protein extracts of IMR90 ER:RAS cells 5 days after 4OHT induction. (I) Representative IF images of IL-8 8 days after 4OHT induction. Scale bar, 100 μm. See also Figure S7 and Tables S2 and S3.

PTBP1 is an RNA binding protein whose best-characterized function is to regulate alternative splicing (Xue et al., 2009). We analyzed alternative splicing during OIS taking advantage of RNA sequencing (RNA-seq) and multivariate analysis of transcript splicing (Shen et al., 2014). We identified 434 splicing events significantly altered (showing a splice change of ≥20%) during OIS in IMR90 cells. These included alternative 5′ or 3′ splice sites, mutually exclusive exons, skipped exons, and retained introns (Figures 7A and S7A; Table S2). PCA of exon inclusion levels of previously published datasets (Tasdemir et al., 2016) suggested that alternative splicing occurring in RAS-induced senescence is likely due to both senescence induction and RAS activation, the latter being the strongest contributor (Figure S7B).

Next, we assessed how PTBP1 depletion affected alternative splicing during OIS. Specifically, we examined exon-skipping events, since these are known to be regulated by PTBP1 (Xue et al., 2009) and are the most frequent type of alternative splicing. While increased exon skipping was observed when comparing senescent and normal cells, knockdown of PTBP1 with two independent shRNAs in senescent cells resulted in increased exon inclusion (Figure S7C). This is in line with PTBP1 repressing exon inclusion (Xue et al., 2009). In addition, we detected a significant enrichment of PTBP1 RNA binding motifs around the splice acceptor site upstream of exons whose inclusion increased with PTBP1 depletion (Figures 7B and S7D), suggesting that those events were directly regulated by PTBP1.

To investigate how alternative splicing mediated by PTBP1 affects SASP regulation, we selected the top 95 genes alternatively spliced in a PTBP1-dependent manner (Figure 7C and Table S3). To determine the alternative splicing events responsible for the altered SASP caused by PTBP1 knockdown, we devised a multi-step screening approach (Figure 7D). First, an siRNA library targeting the 95 candidates regulated by PTBP1 was screened for SASP regulators as previously (Figure 1). The screen identified 13 genes whose knockdown affected IL-8 and IL-6 expression during OIS (Figure 7E). Next, we confirmed by qRT-PCR that the splicing of 8 out of those 13 genes depended on PTBP1 (Figures S7E and S7F). Interestingly, the alternative splicing of five of these genes changed during OIS (e.g., *MARK3*), while this was not the case for the others (e.g., *EXOC7*, Figures S7E and S7F). To verify that a PTBP1-dependent switch in alternative splicing was responsible for regulating the SASP, we designed steric hindrance antisense oligonucleotides (AONs) to target the splicing events, i.e., reverting the inclusion caused by PTBP1 depletion (Figures 7F, S7E, and S7F). Most of the AONs tested partially rescued the downregulation of IL-8 and IL-6 caused by PTBP1 knockdown (Figure 7G).

Two of the genes whose splicing most significantly affected SASP regulation were *EXOC7* and *SNX14*, both involved in regulating different aspects of intracellular trafficking. In particular, EXOC7 is one of the eight core subunits of the exocyst complex that tethers post-Golgi vesicles to the plasma membrane, mediating exocytosis (Wu and Guo, 2015). PTBP1 knockdown regulated the switching between the “long” EXOC7 isoform (EXOC7-L, including exon 7) and the “short” EXOC7 isoform (EXOC7-S, lacking exon 7), and this could be partially prevented using two different AONs (Figures 7H, S7F, and S7G). Restoring the levels of the EXOC7-S isoform using AONs resulted in a partial rescue of IL-6 and IL-8 levels (Figures 7I and S7H). Moreover, publicly available CLIP-seq (crosslinking immunoprecipitation sequencing) data showed that PTBP1 binds near the splice acceptor site upstream of *EXOC7* exon 7 (Figure S7I). Although this suggests that PTBP1 directly controls EXOC7 splicing, we cannot exclude an indirect effect. In summary, these results showed that PTBP1 affects the SASP by controlling the splicing of multiple targets, among them EXOC7.

### PTBP1 Regulates Alternative Splicing of EXOC7 to Control the SASP

**Figure 8:**
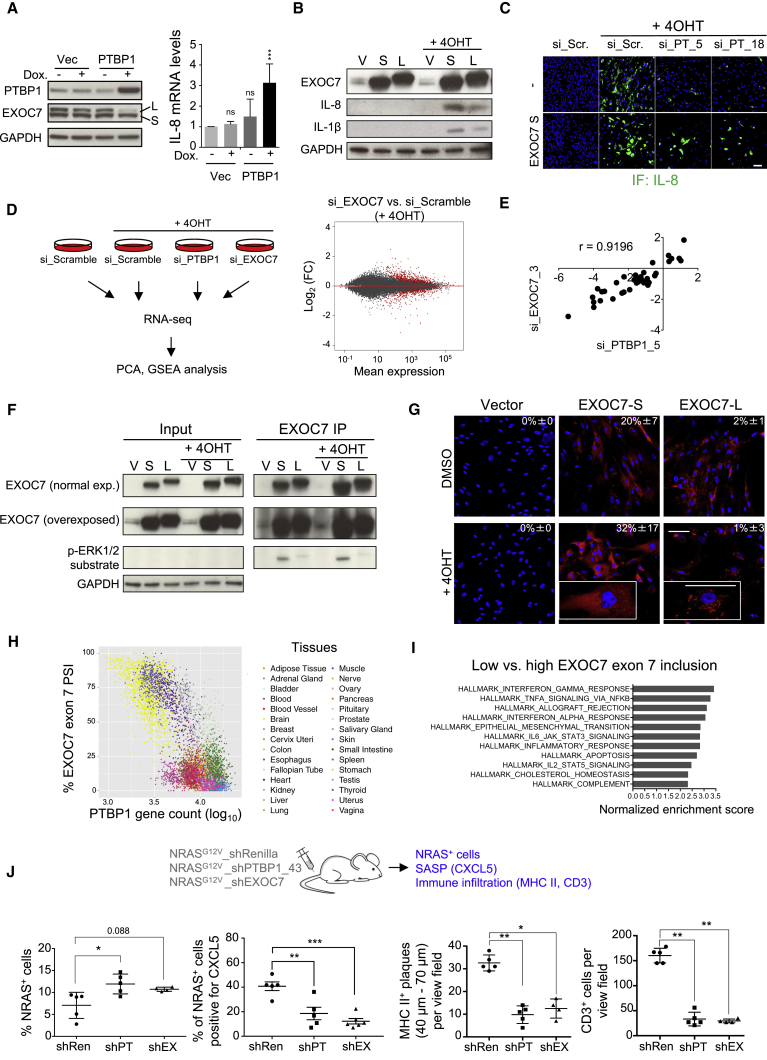
PTBP1 Regulates Alternative Splicing of EXOC7 to Control the SASP (A) SASP expression and EXOC7 isoform switching following PTBP1 overexpression in IMR90 cells. Immunoblot of protein extracts (left) and mRNA analysis by qRT-PCR (right) 2 days after induction of PTBP1 expression with doxycycline (Dox). Normalized and compared with Vec − Dox. Data represent mean ± SD (n = 5). ^∗∗∗^p < 0.001; ns, not significant; one-way ANOVA (Dunnett's test). (B) Comparison of SASP production following overexpression of EXOC7-S (S) and EXOC7-L (L) 4 days after 4OHT and doxycycline treatment of IMR90 ER:RAS cells by immunoblot analysis. v, empty vector. (C) Effect of EXOC7-S on the SASP downregulation caused by PTBP1 knockdown. Representative IF images of IL-8 of IMR90 ER:RAS cells without (−) and with doxycycline treatment (EXOC7 S) 8 days after 4OHT induction. Scale bar, 100 μm. (D and E) Effect of EXOC7 depletion on the SASP (D). Left: experimental design. Right: mean expression (average of the normalized read counts for 3 replicates) in relation to log_2_(FC) for the indicated comparison. Significantly changing genes are highlighted in red. (E) Correlation between the expression of SASP genes upon PTBP1 and EXOC7 siRNA-mediated knockdown. (F) Comparison of EXOC7-S and EXOC7-L phosphorylation assessed by EXOC7 immunoprecipitation followed by immunoblotting. Experimental details as in (B). (G) Comparison of EXOC7-S and EXOC7-L localization to the plasma membrane in proliferating and senescent cells. Quantification of cells showing the diffuse EXOC7 pattern. Data represent mean ± SD (n = 3). p < 0.01, comparing EXOC7-S + DMSO with either Vector + DMSO or EXOC7-L + DMSO; p < 0.05, comparing EXOC7-S + 4OHT with either Vector + 4OHT or EXOC7-L + 4OHT; two-way ANOVA (Bonferroni’s test). Experimental details as in (B). Scale bar, 100 μm. (H) PTBP1 expression versus EXOC7 exon 7 inclusion in data from the Genotype-Tissue Expression (GTEx) project. (I) Top 11 hallmarks with normalized enrichment score >2 and false discovery rate <0.05 in genes with expression positively correlating with EXOC7 exon 7 skipping in GTEx samples. (J) Effect of EXOC7 knockdown on the immune surveillance response. Top: experimental design. Bottom: quantification of NRAS^+^ mouse hepatocytes, CXCL5 expression in NRAS^+^ hepatocytes, and infiltrated MHC II^+^ and CD3^+^ cells 6 days after transposon delivery of NRAS^G12V^_shRenilla (n = 5), NRAS^G12V^_shPTBP1 (n = 4), or NRAS^G12V^_EXOC7 (n = 4). Data represent mean ± SD. ^∗^p < 0.05, ^∗∗^p < 0.01, ^∗∗∗^p < 0.001. Comparisons with NRAS^G12V^_shRenilla, one-way ANOVA (Bonferroni’s test). See also Figure S8.

Extending the OIS observations, knockdown of PTBP1 also affected splicing of EXOC7 in other senescence types such as doxorubicin-induced senescence (Figure S8A). Conversely, overexpression of PTBP1 resulted in preferential expression of the EXOC7-S isoform and SASP induction (Figures 8A and S8B). To understand how EXOC7 isoform switching affects SASP regulation, we ectopically expressed either EXOC7-L (induced by depletion of PTBP1) or EXOC7-S in proliferating and senescent IMR90 cells. In agreement with our results above, senescent cells overexpressing EXOC7-L displayed lower SASP than cells overexpressing EXOC7-S (Figure 8B). Moreover, expression of the SASP-promoting EXOC7-S isoform was sufficient to partially rescue the inhibition of IL-8 and IL-6 expression caused by PTBP1 knockdown (Figures 8C and S8C).

To examine how and to what extent EXOC7 regulates the SASP, we compared the transcriptome of senescent cells lacking PTBP1 or EXOC7 (Figures 8D and S8D). PCA showed a separation between normal and senescent cells, as defined by PC1. Interestingly, depletion of PTBP1 or, to a lesser extent, EXOC7 separated the transcriptomes from those of senescent cells (Figure S8D, right). GSEA analysis showed that, similar to PTBP1 depletion, EXOC7 knockdown was associated with downregulation of SASP and NF-κB-dependent signatures (Figure S8E). Moreover, there was a strong correlation between the effects of EXOC7 depletion and PTBP1 depletion on downregulating specific components of the SASP (Figure 8E).

Since EXOC7 depletion resulted in a reduced SASP, the effect that EXOC7 splice switching has on SASP expression could be due to differential activity. EXOC7 phosphorylation by ERK1/2 regulates exocyst assembly and activity at the plasma membrane (Ren and Guo, 2012). Although ERK1/2 phosphorylation sites are not encoded within exon 7, we observed an increased ERK1/2-mediated phosphorylation of the EXOC7-S isoform (Figures 8F and S8F) that correlated with a distinct subcellular localization of each isoform (Figure 8G). These results suggest that expression of the EXOC7-S isoform favors EXOC7 phosphorylation and membrane localization, corresponding to increased SASP production.

To investigate whether there is a relationship between PTBP1 expression and EXOC7 splicing *in vivo*, we analyzed available gene expression data of human tissues (GTEx) (GTEx Consortium, 2015). *PTBP1* expression levels inversely correlated with inclusion of *EXOC7* exon 7 across different tissues (Figure 8H). High PTBP1 expression and EXOC7 exon 7 skipping were associated with multiple inflammation-related signatures as well as the epithelial-to-mesenchymal transition (Figures 8I and S8G). Together these results suggest that PTBP1-driven alternative splicing of EXOC7 can regulate inflammation in high-turnover tissues *in vivo*.

Finally, we investigated whether EXOC7 can regulate SASP-mediated phenotypes *in vivo*. To this end, we transduced mice with vectors co-expressing oncogenic NRAS (NRAS^G12V^) and a control shRNA or shRNAs targeting PTBP1 or EXOC7 (Figures 8J [top] and S8H). Knockdown of PTBP1 or EXOC7 did not affect senescence (Figure S8I) but resulted in increased numbers of NRAS^+^ hepatocytes (Figure 8J, bottom left). Similar to what we observed upon PTBP1 knockdown, EXOC7 depletion in senescent cells affected the SASP, as exemplified by reduced expression of CXCL5 in NRAS^G12V^-expressing hepatocytes (Figures 8J [second panel from left] and S8J). The formation of macrophage aggregates (MHC II staining) and the infiltration of T cells (CD3 staining) were significantly reduced in the livers of mice co-expressing NRAS^G12V^ and an EXOC7-targeting shRNA (Figures 8J [right panels] and S8K). In conclusion, alternative splicing of EXOC7 contributes to PTBP1-mediated control of the SASP.

## Discussion

Although senescence protects against tumor initiation and limits fibrosis, the aberrant presence of senescent cells can exacerbate age-related pathologies and cancer progression. Consequently, there is growing interest in finding pharmacological agents that suppress the deleterious effects of senescent cells. Until now, studies have concentrated on identifying “senolytic” compounds: drugs that specifically kill senescent cells. Although still ill defined, the current view holds that the SASP is responsible for many of the detrimental effects caused by senescent cells in disease. Thus, SASP inhibition has been proposed as an alternative to senolytics for targeting the harmful effects of senescence. With this in mind, we carried out a systematic search for SASP regulators and found 50 potential therapeutic targets whose inhibition blunts the SASP without posing a risk of bypassing the tumor-suppressive growth arrest. The newly identified SASP regulators differentially modulate the various subsets of the SASP, providing us with a toolbox for ad hoc SASP regulation in future studies.

We focused on one of the screen candidates, the alternative splicing factor PTBP1. Expression of PTBP1 positively correlates with growth of various cancers and poor prognosis (Wang et al., 2017) but had yet to be causally linked to the negative effect of inflammation on advanced cancer. Here, we showed that depletion of PTBP1 inhibited a pro-inflammatory SASP subset without blunting growth arrest or other phenotypes associated with senescence. Hence, PTBP1 presents a potentially powerful therapeutic target for inflammation-driven cancer. Future studies will evaluate whether it could also be used during therapy-induced senescence or age-related disease.

One of our key findings was that PTBP1 depletion prevented the tumor-promoting effects of the SASP. Knocking down PTBP1 prevented tumor growth caused by the presence of senescent cells in two tumor models. A potential caveat of targeting the SASP as a tumor therapy is that it results in decreased clearance of preneoplastic cells by the immune system. However, we did not observe an increased risk of tumorigenesis upon PTBP1 depletion despite the reduced immune surveillance. This could be explained by PTBP1 depletion not inducing cell cycle re-entry and, if anything, exacerbating the senescence growth arrest. This constitutes evidence in a preclinical model that targeting the SASP can be a viable and safe therapeutic strategy in the context of chronic liver disease. Based on the current understanding on how the SASP contributes to different pathologies, PTBP1 inhibition or other anti-SASP therapies could also be used to ameliorate the detrimental effects of chemotherapy or to treat age-related pathologies.

The main role of PTBP1 is to regulate alternative splicing by inducing exon skipping. A three-step screen approach suggested that PTBP1 regulates the SASP by controlling splicing of a number of genes, including EXOC7. EXOC7 is part of the exocyst complex and has been implicated in many cellular features such as neurite outgrowth, epithelial cell polarity, cell motility, or cell morphogenesis (Wu and Guo, 2015). In this study, we demonstrated how EXOC7 also regulates SASP induction. Although the role of the exocyst complex in senescence is still unclear, we postulate that regulation of EXOC7 splicing can affect exocyst activity and can be exploited as a strategy to repress the SASP. Targeting alternative splicing in disease is gaining traction. Strategies employing CRISPR/Cas9 or administration of AONs to induce splice switching have been successful in improving muscular dystrophy and spinal muscular atrophy both in mouse models and in clinical trials (Nelson et al., 2016, Wan and Dreyfuss, 2017).

In summary, we identified 50 genes whose knockdown specifically inhibits the SASP without affecting the senescence growth arrest. One of those genes was PTBP1, a regulator of alternative splicing. Validating the rationale of our screen, knockdown of PTBP1 suppresses the tumor-promoting effects of the SASP without reverting growth arrest in a preclinical model of advanced liver cancer, suggesting that SASP modulation can be a safe way to target inflammation-driven cancers.

## STAR★Methods

### Key Resources Table

**Table:** 

REAGENT or RESOURCE	SOURCE	IDENTIFIER
**Antibodies**		

Rabbit polyclonal anti-53BP1	Novus Biologicals	Cat#NB100-304; RRID: AB_10003037
Mouse monoclonal anti-BrdU (clone: 3D4)	BD Biosciences	Cat#555627; RRID: AB_395993
Mouse monoclonal anti-CCL2/MCP-1 (clone: 24822)	R&D	Cat#MAB279; RRID: AB_2071645
Mouse monoclonal anti-CCL20/MIP-3 alpha (clone 67310)	R&D	Cat#MAB360; RRID: AB_2275415
Mouse monoclonal anti-Exoc7 (clone: D6)	Santa Cruz	Cat#sc-365825; RRID: AB_10843358
Rabbit polyclonal anti-Exoc7	Bethyl	Cat#A303-365A; RRID: AB_10953161
Rabbit polyclonal anti-GAPDH	Abcam	Cat#ab22555; RRID: AB_447153
Purified goat polyclonal anti-CXCL1/GRO alpha	R&D	Cat#AF-275; RRID: AB_355288
Purified goat polyclonal anti-IL-6	R&D	Cat#AB-206-NA; RRID: AB_354281
Mouse monoclonal anti-CXCL8/IL-8 (clone: 6217)	R&D	Cat#MAB208; RRID: AB_2249110
Mouse monoclonal anti-IL-1 alpha/IL-1F1 (clone: 4414)	R&D	Cat#MAB200; RRID: AB_2295862
Mouse monoclonal anti-IL-1 beta/IL-1F2 MAb (clone 8516)	R&D	Cat#MAB201; RRID: AB_358006
Mouse monoclonal anti-p16^INK4a^ (clone: JC-8)	CRUK	N/A
Rabbit polyclonal anti-p21^Cip1^ (M-19)	Santa Cruz	Cat#sc-471; RRID: AB_632123
Mouse monoclonal anti-p53 (clone DO-1)	Santa Cruz	Cat#sc-126; RRID: AB_628082
Rabbit monoclonal anti-phospho-MAPK/CDK Substrates (clone: 34B2)	Cell Signaling Technologies	Cat#2325; RRID: AB_331820
Goat polyclonal anti-PTBP1	Abcam	Cat#ab5642; RRID: AB_305011
Mouse monoclonal anti-Vegfc (clone: 23410)	R&D	Cat#MAB2931; RRID: AB_2212835
Mouse monoclonal anti-phospho-histone H2A.X (clone: jbw301)	Millipore	Cat#05-636; RRID: AB_309864
Donkey anti-goat IgG (H+L), AlexaFluor® 594, conjugated	Thermo Fischer Scientific	Cat#A11058; RRID: AB_142540
Goat anti-mouse IgG (H+L), AlexaFluor® 488, conjugated	Thermo Fischer Scientific	Cat#A11029; RRID: AB_2534088
Rabbit anti-mouse IgG (H+L), AlexaFluor® 488, conjugated	Thermo Fischer Scientific	Cat#A11059; RRID: AB_2534106
Goat anti-mouse IgG (H+L), AlexaFluor® 594, conjugated	Thermo Fischer Scientific	Cat#A11032; RRID: AB_2534091
Goat anti-rabbit IgG (H+L), AlexaFluor® 594, conjugated	Thermo Fischer Scientific	Cat#A11037; RRID: AB_2534095
Goat anti-rat IgG (H+L), AlexaFluor® 488, conjugated	Thermo Fischer Scientific	Cat#A11006; RRID: AB_2534074
Donkey anti-goat IgG-HRP	Santa Cruz	Cat#sc-2020; RRID: AB_631728
Goat anti-mouse IgG-HRP	Santa Cruz	Cat#sc-2005; RRID: AB_631736
Goat anti-rabbit IgG-HRP	Santa Cruz	Cat#sc-2004; RRID: AB_631746
Rat monoclonal anti-MHC Class II (clone: M5/114.15.2)	Novus Biologicals	Cat#NBP1-43312; RRID: AB_10006677
Rabbit polyclonal anti-CD3	Zytomed	Cat#RBK024
Rat monoclonal anti-F4/80 (clone: BM8)	BioLegend	Cat#123105; RRID: AB_893499
Monoclonal anti-mouse Gr-1 (clone: RB6-8C5)	Thermo Fischer Scientific	Cat#11-5931-82; RRID:AB_465314
Monoclonal anti-mouse CD11b (clone: M1/70)	Thermo Fischer Scientific	Cat# 50-0112-82; RRID:AB_11218507
Monoclonal anti-mouse Ly6C (clone: HK1.4)	Thermo Fischer Scientific	Cat#17-5932-82; RRID:AB_1724153

**Chemicals**, **Peptides**, **and Recombinant Proteins**		

(+)-JQ1, BET bromodomain inhibitor	Tocris	Cat#4499; CAS: 1268524-70-4
BW-B 70C, 5-lipoxygenase inhibitor	Tocris	Cat#1304; CAS: 134470-38-5
FIPI, Phospholipase D inhibitor	Tocris	Cat#3600; CAS: 939055-18-2
PD98059, MEK inhibitor	CALBIOCHEM	Cat#513000; CAS: 167869-21-8
Tautomycetin, Protein phosphatase 1 inhibitor	Tocris	Cat#2305; CAS: 119757-73-2
Torin 1, mTOR inhibitor	Tocris	Cat#4247; CAS: 1222998-36-8
XE 991 dihydrochloride, Kv7 voltage-gated potassium channels inhibitor	Tocris	Cat#2000; CAS: 122955-13-9

**Critical Commercial Assays**		

Human IL-6 Quantikine ELISA Kit	R&D	D6050
Human IL-8/CXCL8 Quantikine ELISA Kit	R&D	D8000C
Smart-seq 2	Picelli et al., 2014	N/A
RNAscope® 2.5 Assay HD Reagent Kit-BROWN	Advanced Cell Diagnostics	Cat#322300
RNAscope ® 2.5 LS Probe- Mm-Cxcl5	Advanced Cell Diagnostics	Cat#467441
RNAscope® Positive Control Probe - Mm-Ppib	Advanced Cell Diagnostics	Cat#322300
RNAscope® Negative Control Probe - DapB	Advanced Cell Diagnostics	Cat#310043

**Deposited Data**		

Raw and analyzed data on RNA-Seq for PTBP1 and EXOC7 depletion	This paper	GSE101763
Raw and analyzed data on RNA-Seq for differential regulation of SASP	This paper	GSE101766
GTEx Portal	GTEx Consortium, 2015	N/A
DbGaP	phs000424.v6.p1	N/A
Experimentally determined list of SASP components	Acosta et al., 2013	N/A
PTBP1 CLiP-seq data on 293T cells	Raj et al., 2014	GSE57278
PTBP1 CLiP-seq data on HeLa cells	Coelho et al., 2016 and Xue et al., 2009	E-MTAB-3108 and GSE19323/GSE42701

**Experimental Models**: **Cell Lines**		

IMR90 (human)	ATCC	CCL-186
HFFF2 (human)	ECACC	86031405
MCF-7Tet-Off (human)	BD Biosciences	630907
MEFs (mouse)	Tordella et al., 2016	N/A
5PT (human)	Bauer et al., 2005	N/A
RIL175 expressing luciferase (mouse)	Eggert et al., 2016	N/A

**Experimental Models**: **Organisms/Strains**		

Mouse: C57BL/6	Charles River, Sulzfeld, Germany	N/A
Mouse: B6.129S7-RAG1^tm1Mom^/J (Rag^1-/-^)	The Jackson Laboratory	Stock#002216

**Oligonucleotides**		

‘Human Druggabe Genome’ siRNA library	QIAGEN	HsDgV4, HsGpcrV4.1, HsKinV4.1 and HsPhosV4.1
si_RELA	QIAGEN	SI02663094
si_CEBPB	QIAGEN	SI02777292
si_MAPK14	QIAGEN	SI03089989
si_p53	QIAGEN	SI00011655
si_p16	QIAGEN	SI02664403
si_PTBP1_2	QIAGEN	SI00043631
si_PTBP1_5	QIAGEN	SI00141638
si_PTBP1_18	QIAGEN	SI02649206
si_EXOC7_3	QIAGEN	SI00381787
si_EXOC7_4	QIAGEN	SI00381794
AON Sequences, see Table S4	This paper	N/A
Primer sequences, see Table S5	This paper	N/A

**Recombinant DNA**		

pLNC ER:RAS^V12^	Acosta et al., 2008 and Barradas et al., 2009	Plasmid #67844
Pool of pGIPZ-based shRNAs targeting Brd8	Sigma-Aldrich and MRC LMS Genomics core facility	V3LHS_379314, V3LHS_392041, V3LHS_379315 and V3LHS_379312
Pool of pGIPZ-based shRNAs targeting PP1A	Sigma-Aldrich and MRC LMS Genomics core facility	V2LHS_262414, V3LHS_635633, V3LHS_635634 and V3LHS_635636
Pool of pGIPZ-based shRNAs targeting PTBP1	Sigma-Aldrich and MRC LMS Genomics core facility	V3LHS_640391, V3LHS_645699, V3LHS_362179 and V3LHS_362177
Pool of pGIPZ-based shRNAs targeting PTPN14	Sigma-Aldrich and MRC LMS Genomics core facility	V2LHS_70672, V2LHS_250104, V3LHS_378143 and V3LHS_378142
Pool of pGIPZ-based shRNAs targeting SKP1A	Sigma-Aldrich and MRC LMS Genomics core facility	V2LHS_153803, V2LHS_202391, V2LHS_203112 and V2LHS_202737
Pool of pGIPZ-based shRNAs targeting TMEM219	Sigma-Aldrich and MRC LMS Genomics core facility	V2LHS_180141, V3LHS_392882, V3LHS_392883 and V3LHS_392881
pGIPZ-based shRNA targeting p53	Sigma-Aldrich and MRC LMS Genomics core facility	V3LHS_333920
pGIPZ-based shRNA targeting mTOR	Sigma-Aldrich and MRC LMS Genomics core facility	V3LHS_312661
pGIPZ-based shPTBP1_53	Sigma-Aldrich and MRC LMS Genomics core facility	V3LHS_640391
pGIPZ-based shPTBP1_86	Sigma-Aldrich and MRC LMS Genomics core facility	V3LHS_645699
pCANIG mirE-based shPtbp1.43	This paper	N/A
pCANIG mirE-based shPtbp1.891	This paper	N/A
pCANIG mirE-based shExoc7	This paper	N/A
pTR-mCMV-copGFP	Natarajan et al., 2014	N/A
pTR-NF-κB-mCMV-copGFP	Natarajan et al., 2014	N/A
p95HER2/611	Joaquin Arribas Laboratory; Pedersen et al., 2009	N/A
Tet-ON/PTBP1	This paper	N/A
Tet-ON/EXOC7	This paper	N/A
pLenti-CMV rtTA3	Addgene	w785-1

**Software and Algorithms**		

B-score	Pelz et al., 2010	http://web-cellhts2.dkfz.de/cellHTS-java/cellHTS2/
NPI	Pelz et al., 2010	http://web-cellhts2.dkfz.de/cellHTS-java/cellHTS2/
GraphPad PRISM 7	N/A	https://www.graphpad.com/scientific-software/prism/
TopHat version 2.0.11	Kim et al., 2013	
R version 3.0.1 and 3.2.3		
GSEA version 2.0.12	Liberzon et al., 2015 and Subramanian et al., 2005	http://software.broadinstitute.org/gsea/msigdb
DESeq2 version 1.10.1	Love et al., 2014	https://bioconductor.org/packages/release/bioc/html/DESeq2.html
MATS	Shen et al., 2014	http://rnaseq-mats.sourceforge.net
Maxquant version 1.5.3.8	Cox and Mann, 2008	http://www.coxdocs.org/doku.php?id=maxquant:common:download_and_installation
MeV	Howe et al., 2011	http://mev.tm4.org/
CLIPdb	Yang et al., 2015	N/A
limma	Ritchie et al., 2015 and Phipson et al., 2016	N/A
IGV Browser	Robinson et al., 2011	http://software.broadinstitute.org/software/igv/download
UCSC Genome Browser	Kent et al., 2002	https://genome.ucsc.edu/cgi-bin/hgGateway?redirect=manual&source=genome.ucsc.edu

### Contact for Reagent and Resource Sharing

Further information and requests for resources and reagents should be directed to and will be fulfilled by the Lead Contact, Jesús Gil (jesus.gil@imperial.ac.uk)

### Experimental Model and Subject Details

#### Cell Lines

IMR90, MCF7 Tet-Off and HFFF2 were cultured in DMEM (Gibco) supplemented with 10% fetal bovine serum (Sigma) and 1% antibiotic-antimycotic solution (Gibco). MEFs were cultured in GMEM (Sigma) and 1% antibiotic-antimycotic solution (Gibco). HNSCC cell line 5PT was cultured in keratinocyte growth medium (KGM).

To generate IMR90 cells expressing ER:RAS^G12V^, miR30-based shRNAs (pGIPz) or κB-GFP (pTR-mCMV-copGFP), and MEFs expressing miRE-based shRNAs, retroviral and lentiviral infections were carried out as previously described (Aarts et al., 2017). To generate IMR90 ER:RAS^G12V^ cells expressing two miR30-based shRNAs simultaneously, viruses were mixed at a 1:1 ratio. To generate MCF7 Tet-Off cells expressing the carboxy-terminal fragment of HER2, known as ‘p95HER2’ or ‘611CTF’, under the control of a Tet-responsive element, MCF7 cells were infected with the appropriate viruses. To generate IMR90 PTBP1 Tet-ON or IMR90 ER:RAS EXOC7 Tet-ON, cells were infected with equal amounts of iCMV-tight and rtTA3 viruses. To select cells that efficiently integrated both constructs the cells were treated with 0.8 μg/ml Puromycin and 25 μg/ml Hygromycin.

To induce OIS, IMR90 ER:RAS cells were treated with 100 nM 4-hydroxytamoxifen (4OHT; Sigma) reconstituted in DMSO or MCF7 Tet-Off/p95HER2 were depleted of 1 μg/ml doxycycline (dox; Sigma). To induce chemotherapy-induced senescence, IMR90 cells were treated with 0.4 μM doxorubicin (Sigma) for 24 hr which was subsequently removed by media change until analysis timepoint. To induce expression of PTBP1 or EXOC7, cells were treated with 100 and 12 ng/ml Doxycyline (Sigma), respectively, added on the day of 4OHT induction.

#### Mice

For xenograft experiments, 1x10^6^ 5PT cells ± 3x10^6^ IMR-90 were re-suspended in 150ml of serum free DMEM; 100ml of this mix was injected subcutaneously into the flank of partially immunocompromised B6.129S7-*Rag1*^*tm1Mom*^/J (*Rag1*^*-/-*^) female mice (5-6 months old) as previously described (Herranz et al., 2015). Tumor size was measured using an electronic calliper and calculated using the formula 4π/3 x r^3^ (radius (r) calculated from the average diameter, measured as the tumor width and length). Optical imaging was performed using the IVIS SpectrumCT system. All animal work followed institutional guidelines of the University of Southampton (UK) and has been approved by UK legal authorities.

For hepatocyte senescence experiments, 4-6-week old female C57BL/6 mice were purchased from Charles River (Sulzfeld, Germany). Intrahepatic delivery of a transposon-based plasmid pCaNIG-shRNA allowing co-expression of NRAS^G12V^ and miR30-based shRNAs together with an expression plasmid for sleeping beauty 13 was performed via hydrodynamic tail vein injection (HDTV) as described previously (Kang et al., 2011). For the short-term tumorigenesis study, seeding of luciferase-expressing hepatocellular carcinoma RIL175 cells in senescent livers 4 days post-HDTV was achieved by intrasplenic injection as previously described (Eggert et al., 2016). For immune surveillance analysis by immunohistochemistry, mice were sacrificed 6 days after HDTV and livers were collected and either 10% formalin-fixed, paraffin embedded, or embedded in OCT compound (Tissue-Tek) and frozen. For tumorigenesis assessment 15 days after RIL175-cell seeding, livers were explanted and incubated with firefly luciferin (Biosynth) at a concentration of 0.381 mg/ml for 10 min at room temperature. Livers were consequently imaged with AEQUORIA MDS (Hamamatsu Photonics, Hamamatsu, Japan). In the long-term study, the livers were monitored by ultrasound and mice were sacrificed when termination criteria were fulfilled. All animal work followed institutional guidelines of Tübingen University and has been approved by German legal authorities.

### Method Details

#### Vector Construction

pLNC-ER:RAS has been previously described (Acosta et al., 2008). pGIPZ-based shRNA vectors were obtained from Sigma. pTR-mCMV-kB-copGFP and empty vector were a gift from Venkatesh Natarajan. To generate the pCANIG-mirE-based shRNA targeting mouse PTBP1, first the miR30-based shRNA from the pGIPZ vector V3LMM_509343 was converted to miRE-based shRNA by PCR amplification using the primers miRE-Xho-short-fw and miRE-EcoPlasmid-Rev. For *de novo* generation of miRE-based shRNAs, the 97-mer oligonucleotides mousePTBP1.891 and mouseEXOC7.1546 were PCR amplified using the primers miRE-Xho-fw and miRE-EcoOligo-rev and cloned into the pRRL lentiviral backbone SGEP. The miRE-based shRNAs were then shuttled into the transposon plasmid pCaNIG-shRNA using XhoI and MluI fragments. 97-mers were as follows:

**Table:** 

PTBP1.891:	TGCTGTTGACAGTGAGCGACAGTCTCAATGTCAAGTACAATAGTGAAGCCACAGATGTATTGTACTT GACATTGAGACTGGTGCCTACTGCCTCGGA
EXOC7.1546:	TGCTGTTGACAGTGAGCGACGCCATCTTCCTACACAACAATAGTGAAGCCACAGATGTATTGTTGTG TAGGAAGATGGCGCTGCCTACTGCCTCGGA

To generate a Tet-ON/PTBP1 expressing vector, first, full-length cDNA encoding PTBP1 (NM_002819.4) was PCR amplified from pBABE-PTBP1 vector custom-synthesised by GenScript, using primers CMVPTBP1F (5’-CGTTCGAAGCCACCATGGACGGCATTGTCCCA-3’) and CMVPTBP1R (5’-CCGGTTTAAACCTAGATGGTGGACTTGGAGAAG-3’), and the Platinum® PCR SuperMix High Fidelity (Invitrogen) according to manufacturer’s instructions. The PTBP1 amplicon was then shuttled into the pLenti-CMV-tight inducible (iCMV-tight) vector using BstBI and PmeI fragments. A MCS linker had previously been introduced in place of the eGFP in the iCMV-tight vector. The iCMV-tight and the pLenti-CMV-rtTA3 (reverse tetracycline controlled transactivator) plasmids were purchased from Addgene (w771-1 and w785-1, respectively).

To generate a Tet-ON/EXOC7 expressing vector, EXOC7 short and long isoforms were cloned in the iCMV-tight vector. First, EXOC7 short and long isoforms were prepared by PCR amplification using the EXOC7cloningF (5’-CGTTCGAAGCCACCATGGACTACAAGGACGACGATGACAAGATTCCCCCACAGGAG-3’) and EXOC7cloningR (5’-CCGCCTGCAGGTCAGGCAGAGGTGTCGAAAAGGC-3’) primers and as template the cDNA (produced with oligo-dT primers) from IMR90 cells or shPTBP1 IMR90 cells, respectively. The EXOC7 amplicons were then shuttled into the iCMV-tight vector using BstBI and SbfI fragments.

#### Growth Assays

BrdU incorporation and colony formation assays with crystal violet were performed as previously described (Herranz et al., 2015). Briefly, for BrdU incorporation assays, the cells were incubated with 10 μM BrdU for 16-18 hr before being fixed with 4% PFA (w/v). BrdU incorporation was assessed by Immunofluorescence and High Content Analysis microscopy. For crystal violet staining, the cells were seeded at low density on 6-well dishes and maintained for 10-14 days in the absence or presence of 4OHT. Upon colony formation or confluence of a control sample, the cells were fixed with 0.5% glutaraldehyde (w/v). The plates were then stained with 0.2% crystal violet (w/v).

#### Inhibitor Treatments

The working concentrations of chemical compounds were determined after dose-response testing. Specifically, (+)-JQ1 at 40 nM, BW-B 70C at 2 μM, FIPI at 1 μM, PD98059 at 20 μM (Acosta et al., 2013), Tautomycetin at 150 nM, Torin 1 at 25 nM (Herranz et al., 2015) and XE 991 dihydrochloride at 20 μM. All chemical compounds were reconstituted in DMSO. Inhibitor treatment was initiated simultaneously to 4OHT-induction. Drug-containing media was refreshed every 2 days by media change to prevent additive effects.

#### Nucleic Acid Transfections

siRNAs were purchased from Qiagen lyophilised either in a Flexitube® or spotted in Flexiplates®. When available, the experimentally verified siRNA sequences were preferred. For immunofluorescence analysis, IMR90, IMR90 ER:RAS cells, or senescent IMR90 ER:RAS cells in suspension (100 μl) were reverse transfected with siRNAs on a well of a 96-well plate. The suspension media was DMEM supplemented with 10% FBS only. The transfection mix for each sample well contained 0.1 μL DharmaFECT™ 1 (GE Healthcare) in 17.5 μL plain DMEM mixed with 3.6 μL siRNA 30 min prior to cell seeding. 18 hr after transfection, allowing target cells to adhere, the media were replaced with fresh complete media, containing 4OHT when appropriate. The cells were fixed at the specified time-point with 4% PFA (w/v). For mRNA analysis, the procedure was identical but scaled up 20 times to fit a 6-well plate. The cells were harvested by scraping in 0.8 ml TRIzol® RNA isolation reagent (Ambion) per well.

AONs were rationally designed using a computational approach previously described (Pramono et al., 2012), which considers co-transcriptional binding accessibility and relevant putative splice motifs of target, and AON-to-target binding thermodynamics. Sequences of AONs used in this study are provided in Table S4. All the designed AONs were synthesized by IDT as single-stranded RNA bases each modified with 2’-O-methyl and phosphorothioate backbone. Forward transfection of AONs was carried out as per siRNA transfection with the only difference being the addition of transfection mix on adhered cultures plated 3 or 4 days in advance.

#### Conditioned Media (CM) Experiments

IMR90 ER:RAS cells were seeded in a 10 cm dish for each condition. The next day the media were replaced with induction media (4OHT addition or doxycycline removal). For paracrine senescence or SASP analysis by ELISA, the media were replaced 3 days later with DMEM supplemented with 0.5% (v/v) FBS and 1% antibiotic-antimycotic solution. 4 days later, ensuring each 10 cm dish contained confluent but alive cells, the CM were collected and initially centrifuged at 2,500 rpm to remove cellular debris. The CM were then filtered through a 0.2 μm pore size cellulose acetate membrane (Gilson). For paracrine senescence experiments, the resulting media was mixed in a 3 to 1 ratio with DMEM supplemented with 40% (v/v) FBS. For SASP analysis by ELISA, equal volumes of CM were used and assay performed as per manufacturer’s instructions (R&D Systems). The samples were diluted 1,000X and each diluted sample was represented twice on the plate. The absorbance reading was taken at 450 nm (A_595_) in a SpectraMAX340PC (Molecular Devices) microplate reader. Protein concentration was then estimated according to a calibration curve obtained from the absorbance values of a dilution series of the supplied standard protein control.

For proteomic analysis of the secretome, before replacement to appropriate media on the 3rd day, the cells were washed 3 times with pre-warmed PBS. After that, the cells were cultured for another 3 days in high glucose, no glutamine, no phenol red DMEM supplemented with L-Glutamine (Gibco), no FBS and 4OHT where appropriate. On the 6th day, the CM were collected and processed as mentioned above. Then the CM were concentrated by ultracentrifugation using the Vivaspin 20 5 kDa MWCO columns (GE Healthcare) about 100 times (Herranz et al., 2015). At this point, the protein concentration was determined using the Pierce™ BCA Protein Assay Kit (Thermo Scientific). Mass spectrometry of the CM was performed as previously described (Herranz et al., 2015).

#### Total RNA Extraction

Cells were scraped and homogenized in 0.8 ml TRIzol® RNA isolation reagent (Ambion), mixed with 150 μl of Chloroform (Sigma), vortexed for 15 s and centrifuged at 15,000 rpm at 4°C for 30 min. After the phase separation step, the top clear RNA-containing phase was purified using the RNAeasy® Mini Kit (Qiagen) from step 2 onwards according to manufacturer’s protocol. For mouse liver RNA extraction, harvested liver tissues were mixed with Qiazol Lysis Reagent (Qiagen) and homogenized with an electric homogenizer. After adding chloroform samples were centrifuged and the upper phase was mixed with isopropanol to precipitate RNA. Remaining DNA was digested with DNAseI (NEB). RNA was then purified with RNAeasy Mini Kit (Qiagen). RNA concentration was measured using a NanoDrop® ND-1000 UV-Vis spectrophotometer at an absorbance of 260 nm (A260).

#### cDNA Synthesis and Quantitative RT-PCR

cDNA synthesis from cell total RNA was carried out using random hexamers, unless otherwise specified, and the SuperScript® II Reverse Transcriptase (RT) kit (Invitrogen) according to manufacturer’s instructions. cDNA synthesis from mouse total RNA was performed using PrimeScript™ RT Master Mix (Takara, RR036A).

For gene expression analysis, PCR amplification was performed using SYBR® Green PCR Master Mix (Applied Biosystems) and the samples were run on CFX96™ Real-Time PCR Detection system (Bio-Rad). The primers were designed using PrimerBank or Primer-BLAST to span exon-exon junctions, or to flank an intron of > 1 kb in size, to anneal to all transcript variants of the gene of interest, and to generate a PCR product of no more than 150 bp. To calculate gene expression (‘mRNA levels’) the ΔΔCt method was used with the Ribosomal protein S14 (*RPS14*) expression as a normalizer and an untreated sample as relative control.

For alternative splicing analysis, PCR amplification was performed using Platinum® PCR SuperMix High Fidelity (Invitrogen) and the samples were run on a Dyad Peltier Thermal cycler (Bio-Rad). The primers were specifically designed to anneal to flanking exons of the alternatively spliced exon. The PCR product was then size-resolved using Agilent 2100 Bioanalyzer and Agilent High Sensitivity DNA Analysis Kit following manufacturer’s instructions and the bands were visualized using the 2100 Expert Software. In any given sample, PSI (Ψ) was calculated by the given concentration of the larger product (included exon) relative to the sum of concentrations of both products. Sequence of primers used in this study are provided in Table S5.

For mouse SASP analysis, Real-Time-PCR was conducted using the SYBR® Premix Ex Taq™ (Takara, RR420A) with the RT^2^ Profiler™ PCR Array Mouse Cytokines & Chemokines Kit (Qiagen, PAMM-150ZA-12) in a 7300 Real Time PCR machine (Applied Biosystems). Data was analyzed with 7500 software (Applied Biosystems) and Data Analysis Center webtool (Qiagen) with the ΔΔCt method using the mean expression of *Actb*, *Gapdh* and *Hsp90ab1* as normalizers.

#### RNA-Sequencing

The RNA-sequencing libraries for the shPTBP1 and siEXOC7 experiments were prepared as described previously (Tordella et al., 2016) and were run on a Hiseq2500. For alternative splicing analysis (shPTBP1), we obtained on average 71 million 100bp paired end sequencing reads for each replicate of each condition (average of 213 million reads per condition). For GSEA (siEXOC7) we obtained on average 40 million single-end 50 bp reads for each sample.

The RNA-sequencing library for the siRNAs repressing the SASP (2 different siRNAs targeting each one of the 38 genes plus additional controls) was prepared following the Smart-seq2 protocol Picelli et al. (2014). siRNA transfection onto 96-wells was performed as described above and on the day of collection the cells in each well were lysed in 50 μl of lysis buffer which was prepared according to Smart-seq2. 2 μl of lysate was used for the following steps described in detail by (Picelli et al., 2014). The RNA-seq library containing 272 samples was run on a Hiseq2500 using single-end 50-bp reads with a coverage depth of 2.5x10^6^ reads per sample in over 80% of samples.

#### Immunofluorescence Staining of Cells

Cells were grown on 96-well plates, fixed with 4% PFA (w/v) and stained as follows. The cells were permeabilised in 0.2% Triton® X-100 (v/v) (Sigma) diluted in PBS for 10 min, blocked with 1% BSA (w/v) (Sigma) and 0.4% fish gelatin (v/v) (Sigma) for 30 min, treated with primary antibody for 40 min, then with fluorescence labelled secondary antibody (Alexa Fluor®) for 30 min and finally treated with 1 μg/ml DAPI (Sigma) for 10 min. Primary and secondary antibodies were suspended in blocking solution. All incubations were followed by 3 PBS washes. For SA-β-Galactosidase assessment, live cells were treated with 100 μM 9H-(1,3-Dichloro-9,9-Dimethylacridin-2-One-7-yl) β-D-Galactopyranoside (DDAOG, Molecular Probes™) for 2 hr prior to PFA fixation and subsequently stained with 1 μg/ml DAPI (Sigma) for 10 min.

#### Cytochemical SA-β-Galactosidase Assay

Cells were grown on 6-well plates, fixed with 0.5% glutaraldehyde (w/v) (Sigma) in PBS for 10-15 min, washed with 1 mM MgCl_2_ in PBS (pH 6.0) 2-3 times and then incubated with X-Gal staining solution (1 mg/mL X-Gal, Thermo Scientific, 5 mM K_3_[Fe(CN)_6_] and 5 mM K_4_[Fe(CN)_6_]) for 16-18 hr at 37°C with gentle agitation. Bright field images of cells were taken using the DP20 digital camera attached to the Olympus CKX41 inverted light microscope. The percentage of SA-β-Gal positive cells was estimated by counting at least 200 cells per replicate sample facilitated by the ‘point picker’ tool of ImageJ software.

#### Staining of Tissue Sections

Sections (2-4 μm) of paraffin-embedded mouse liver were processed for IF analysis, IHC or RNA *in situ* hybridization. For IF, antigen retrieval was carried out with 10 mM sodium citrate (pH 6) for 20 min in a steamer, after which sections were treated with Protein Block (Dako) for 10 min, incubated with primary antibody O/N at 4°C, incubated with fluorescence labeled secondary antibody (Alexa Fluor®) for 1 hr and finally mounted in FluoroMount-G®. Primary and secondary antibodies were suspended in Antibody Diluent (Dako).

For colorimetric-based IHC, antigen retrieval was carried out with EDTA or sodium citrate buffer, after which sections were incubated with antibodies against antigens in BONDTM primary antibody diluent (AR9352, Leica Biosystems). Primary antibody exposure was followed by secondary antibody (Leica Biosystems) and staining using the Novolink™ DAB (Polymer) kit (RE7230-K, Leica Biosystems). Brightfield images were captured using a Leica DM 1000 LED microscope and processed using Leica Application Suite software (Leica Biosystems).

RNA *in situ* hybridization in 5 μm liver sections was carried out using the RNAscope® 2.5 Assay (FFPE and 2.5 HD Brown Assay) from Advanced Cell Diagnostics, according to manufacturer’s protocol. Probes for *Cxcl5* (Cat#467448), the housekeeping gene *Ppib* (positive control, Cat#313911) and the bacterial gene dapB (negative control, Cat#310043) were purchased from Advanced Cell Diagnostics. Signal detection was carried out by DAB staining. Slides were counterstained with haematoxylin prior to mounting and then whole digital slides were acquired using the Leica SCN400 scanner (Leica) at X20 magnification.

For SA-β-Galactosidase assessment, frozen sections (6 μm) were fixed in ice-cold 0.5% glutaraldehyde (w/v) solution for 15 min, washed 2 times in 1 mM MgCl_2_/PBS (pH 6.0) for 5 min, then incubated with X-Gal staining solution (1 mg/mL X-Gal, Thermo Scientific, 5 mM K3[Fe(CN)_6_] and 5 mM K4[Fe(CN)_6_]) for 16-18 hr at 37°C, washed 2 times in distilled water, counterstained with eosin for 30 s, dehydrated and mounted in VectaMount™. Quantification was performed as previously described (Tordella et al., 2016).

#### High Content Analysis (HCA)

IF imaging was carried out using the automated high-throughput fluorescent microscope IN Cell Analyzer 2000 (GE Healthcare) with a 20x objective with the exception of DNA damage foci analysis which required a 40x objective. Fluorescent images were acquired for each of the fluorophores using built-in wavelength settings (‘DAPI’ for DAPI, ‘FITC’ for AlexaFluor® 488 FITC, ‘Texas Red’ for AlexaFluor® 594 and ‘Cy5’ for DDAOG). Multiple fields within a well were acquired in order to include a minimum of 1,000 cells per sample-well. HCA of the images were processed using the IN Cell Investigator 2.7.3 software as described previously (Herranz et al., 2015). Briefly, DAPI served as a nuclear mask hence allowed for segmentation of cells with a Top-Hat method. To detect cytoplasmic staining in cultured cells, a collar of 7-9 μm around DAPI was applied. To detect cytoplasmic staining in tissue sections, a multiscale top-hat parameter was set on the reference wavelength (typically NRAS staining). Nuclear IF in the reference wavelength, i.e. all the other wavelengths apart from DAPI, was quantitated as an average of pixel intensity (grey scale) within the specified nuclear area. Cytoplasmic IF in the reference wavelength was quantitated as a coefficient of variance (CV) of the pixel intensities within the collar area. Nuclear foci IF in the reference wavelength was quantified as n number of foci per nucleus. In samples of cultured cells, a threshold for positive cells was assigned above the average intensity of unstained or negative control sample unless otherwise specified. In tissue sections, a threshold for positive cells was assigned above background staining using the built-in ‘cell to background ratio’ measurement. Immunohistochemistry imaging and quantification was also automated.

#### FACS

The liver was chopped into small ∼1 mm3 pieces and then enzymatically digested in a medium composed of equal volume of DMEM and HBS supplemented with 0.5 mg/ml Collagenase (Serva Collagenase NB 4G) for 30 min at 37°C. The enzymatic reaction was stopped using cold medium and the liver suspension was meshed through a 70 μm nylon mesh (Falcon). After centrifugation erythrocytes were lysed using an ACK buffer (150 mM NH4Cl, 10 mM KHCO3, and 0.1 mM EDTA). 10^6^ cells were resuspended in blocking solution (2% BSA in PBS) and stained with antibodies on ice for 30 min. Samples were immediately acquired using a FACSCanto flow cytometer (BD Biosciences). Samples were gated on viable leukocytes by DAPI exclusion and doublets were excluded using height versus area dot plots. Gating strategies were described previously (Eggert et al., 2016) and depicted in Figure S6. Data analysis was performed using FlowJo software (Tree Star).

#### Immunoblot and Immunoprecipitation

Cells were lysed in RIPA buffer (80 mM Tris pH 8.0, 150 mM NaCl, 1% Triton® X-100, 0.5% Na-Doc, 0.1% SDS, 1mM EDTA) supplemented with 1 tablet of phosphatase and 1 tablet of protease inhibitors (Roche) per 10 ml RIPA. Lysis was performed on ice for 20 min with occasional vortexing followed by centrifugation at 15,000 rpm at 4°C for 15 min to collect protein extracts. Immunobloting was carried out as previously described (Herranz et al., 2015). Immunoprecipitation was performed by incubating the lysate (equal amounts of protein) with antibody (EXOC7 sc-365825 or control IgG) for 2 hr at 4°C and Dynabeads Protein A for 1 hr at 4°C, before being washed 3 times in RIPA buffer and eluted in Laemmli buffer for 10 min at 95°C.

#### siRNA Screen Analysis

The screen readouts were normalized by B-score and Normalized Percent Inhibition (NPI) using the freely available online software found at http://web-cellhts2.dkfz.de/cellHTS-java/cellHTS2/ (Pelz et al., 2010). For NPI calculation, each sample was normalized to the control siRNAs present in the same 96-well plate. The scramble siRNA transfected cells served as negative controls and the cells transfected with siRNAs targeting RELA and C/EBPβ served as positive controls. K-means clustering was performed using R.

#### Analysis of Proteomics Data

Raw files were analysed using Maxquant (Cox and Mann, 2008). Files were searched against the Swissprot human database (downloaded on August 10^th^ 2015). Protein sequences were reversed to provide a decoy database that enabled a protein and peptide false discovery rate of 1%. Fixed modification of cysteine residues (carbamidomethylated) and variable modification of methionine residues (oxidised) were included. Protein quantification information was produced using the label-free quantification (“LFQ”) algorithm to enable direct comparison of protein intensity between samples. The list of proteins was then filtered down to SASP proteins according to a previously experimentally determined list (Acosta et al., 2013). Protein measurements were then mean normalized using the ‘scale’ function in R and visualized as heatmaps using the unsupervised hierarchical clustering option in MultiExperiment Viewer (MeV).

#### Analysis of RNA Sequencing Data

RNA-seq sequencing reads were aligned to hg19 genome with TopHat using parameters “--library-type fr-firststrand” and using known transcripts annotation from Ensembl gene version 70. Number of reads on exons were summarised using featureCounts function available in rsubread R package (Liao et al., 2013). Differential expression analysis was performed using DESeq2 (Love et al., 2014, Tordella et al., 2016, Trapnell et al., 2009). PCA plots were generated using plotPCA() function from DESeq2 Bioconductor package.

To generate the heatmaps shown in Figures 2D, 2E, and S2D, we initially identified differentially expressed genes between non-senescent conditions (in total 15 replicates) and senescent conditions (in total 6 replicates) using DESeq2 with adjusted p value of 0.05. Differentially expressed genes were filtered for and their normalized expression values (rlog) were obtained from DESeq2. Clustering was performed with heatmap.2 function available in gplots R package version 3.0.1 with Z-score transformed rlog values. Summary heatmap (Figure 2E) was created using average rlog values from gene and shRNA clusters. To determine differential alternative splicing between samples, Multivariate Analysis of Transcript Splicing (MATS) on R (Shen et al., 2014) was used. The source-code is freely available at http://rnaseq-mats.sourceforge.net. The user-defined cut-off used for the likelihood-ratio statistical test was assigned to 20% splicing change. Shashimi plots were generated with IGV.

#### Gene Set Enrichment Analysis (GSEA) of RNA-Seq Data

For each differential expression analysis comparison, genes were ranked using “wald statistics” obtained from DESeq2 and GSEA was carried out on these ranked lists on all curated gene sets available in MSigDB (http://software.broadinstitute.org/gsea/msigdb). DESeq2 independent filtering is based on mean of normalised read counts and filters out genes with very low expression level. The SASP and OIS GSEA signatures were derived from (Acosta et al., 2013) as described before (Herranz et al., 2015, Tordella et al., 2016).

#### RNA-Binding Motif Analysis

FIMO (Grant et al., 2011) was used to scan the human gene sequences for the PTBP1 RNA-binding motifs inferred by (Ray et al., 2013). The thereby predicted occurrences were mapped to the analyzed splicing events. To generate the RNA-maps (Figures 7B and S7D), for each comparison alternative exons were divided into those with PSIs significantly increasing upon PTBP1 knockdown (putatively repressed), those with PSIs significantly decreasing upon PTBP1 knockdown (putatively enhanced), and those with PSIs not altered upon PTBP1 knockdown (putatively not regulated). Statistical significance for local motif enrichment is associated with Fisher’s exact tests for differences in motif occurrences between groups of exons within 31 bp moving windows.

#### CLIP Data Visualization

Publically available pre-processed (i.e. genomically mapped in BED-formatted files) PTBP1 CLIP-Seq data were localized through CLIPdb (Yang et al., 2015). Those included iCLIP and PAR-iCLIP in different samples of 293T cells (GEO accession GSE57278; Raj et al., 2014), and HITS-CLIP on HeLa cells (GEO accessions GSE19323/GSE42701; Xue et al., 2009). A BedGraph formated file of PTBP1 iCLIP data from HeLa cells (ArrayExpress accession E-MTAB-3108; Coelho et al., 2016) was also retrieved. The UCSC Genome Browser (Kent et al., 2002) was used for the visualization (Figure S7I).

#### Cross-Tissue Analysis of PTBP1 Expression and EXOC7 Exon 7 Inclusion

The data used for these analyses were obtained from the GTEx Portal (GTEx Consortium, 2015) and dbGaP (Accession number phs000424.v6.p1 on 2017/09/03). Gene expression was quantified from gene read counts and only genes sufficiently expressed (sum of counts per million (CPM) across all samples > 10) were kept for further analyses. Quantile normalization of gene expression was performed using the *normalizeBetweenArrays* function in *limma* (Ritchie et al., 2015). Splicing of *EXOC7* exon 7 was quantified from the respective exon junction reads, using the percent spliced-in (PSI) metric, as in Barbosa-Morais et al. (2012). For both gene expression and alternative splicing analyses, samples corresponding to patient-derived cell lines and two samples with missing values for *EXOC7* exon 7 skipping quantification were excluded.

#### Cross-Tissue Gene Set Enrichment Analysis

Samples were separated based on their PTBP1 expression levels (cut-off set as the mean PTBP1 expression level across samples, approximately 9005 normalized read counts) or EXOC7 exon 7 inclusion levels (cut-off set as the local density minimum separating the two peaks of the respective bimodal PSI distribution, 0.4). Gene expression was modelled as a function of sample separation according to PTBP1 expression or EXOC7 exon 7 inclusion using *limma*’s *lmFit* (Phipson et al., 2016). GSEA with Hallmark Gene Sets (Liberzon et al., 2015), was run on the list of genes ordered by descending t-statistic for the subsequent differential gene expression analysis.

### Quantification and Statistical Analyses

For the secondary validation siRNA screen, 3 replicate NPI values of each sample siRNA were compared to all scramble siRNA values by unpaired, Student’s t-test. For xenograft experiments, differences between tumor growth rates were tested for statistical significance using a 2-tailed homoscedastic t-test comparing the area under the curve (AUC) for each individual tumor within a treatment group. For the proportions of increased exon skipping/inclusion (Figure S7B), one-sided Pearson's chi-square tests against an expected proportion of 0.5 were used. For alternative splicing analysis by RMATS, splicing changes with a false discovery rate (FDR) less than 0.05 were considered statistically significant. For the rest of cell culture experiments and for mouse experiments, significant differences were determined by one-way ANOVA or two-way ANOVA (for grouped data). Asterisks (^∗^) always indicate significant differences as follows. ns = not significant, ^∗^ = p < 0.05, ^∗∗^ = p < 0.01, ^∗∗∗^ = p < 0.001. n=number of biological replicates, unless otherwise specified.

### Data and Software Availability

RNA-seq data have been deposited at the Gene Expression Omnibus under the accession numbers GSE101763 (comprising GSE101750 and GSE101758) and GSE101766.

## Supplemental Information

Document S1. Figures S1–S8 and Tables S1 and S3–S5

Table S2. Splicing Events Altered during OIS, Related to Figure 7

Document S2. Article plus Supplemental Information
