# Supplementary material for: PTBP1-Mediated Alternative Splicing Regulates the Inflammatory Secretome and the Pro-tumorigenic Effects of Senescent Cells
Source: Cancer Cell. 2018 Jul 9;34(1):85–102.e9. doi: 10.1016/j.ccell.2018.06.007 (PMC6048363; doi:10.1016/j.ccell.2018.06.007)
Supplement: Document S1. Figures S1–S8 and Tables S1 and S3–S5 [file mmc1.pdf]

Cancer Cell, Volume *34*

Supplemental Information

PTBP1-Mediated Alternative Splicing

Regulates the Inflammatory Secretome

and the Pro-tumorigenic Effects of Senescent Cells

Athena Georgilis, Sabrina Klotz, Christopher J. Hanley, Nicolas Herranz, Benedikt Weirich, Beatriz Morancho, Ana Carolina Leote, Luana D'Artista, Suchira Gallage, Marco Seehawer, Thomas Carroll, Gopuraja Dharmalingam, Keng Boon Wee, Marco Mellone, Joaquim Pombo, Danijela Heide, Ernesto Guccione, Joaquín Arribas, Nuno L. Barbosa-Morais, Mathias Heikenwalder, Gareth J. Thomas, Lars Zender, and Jesús Gil

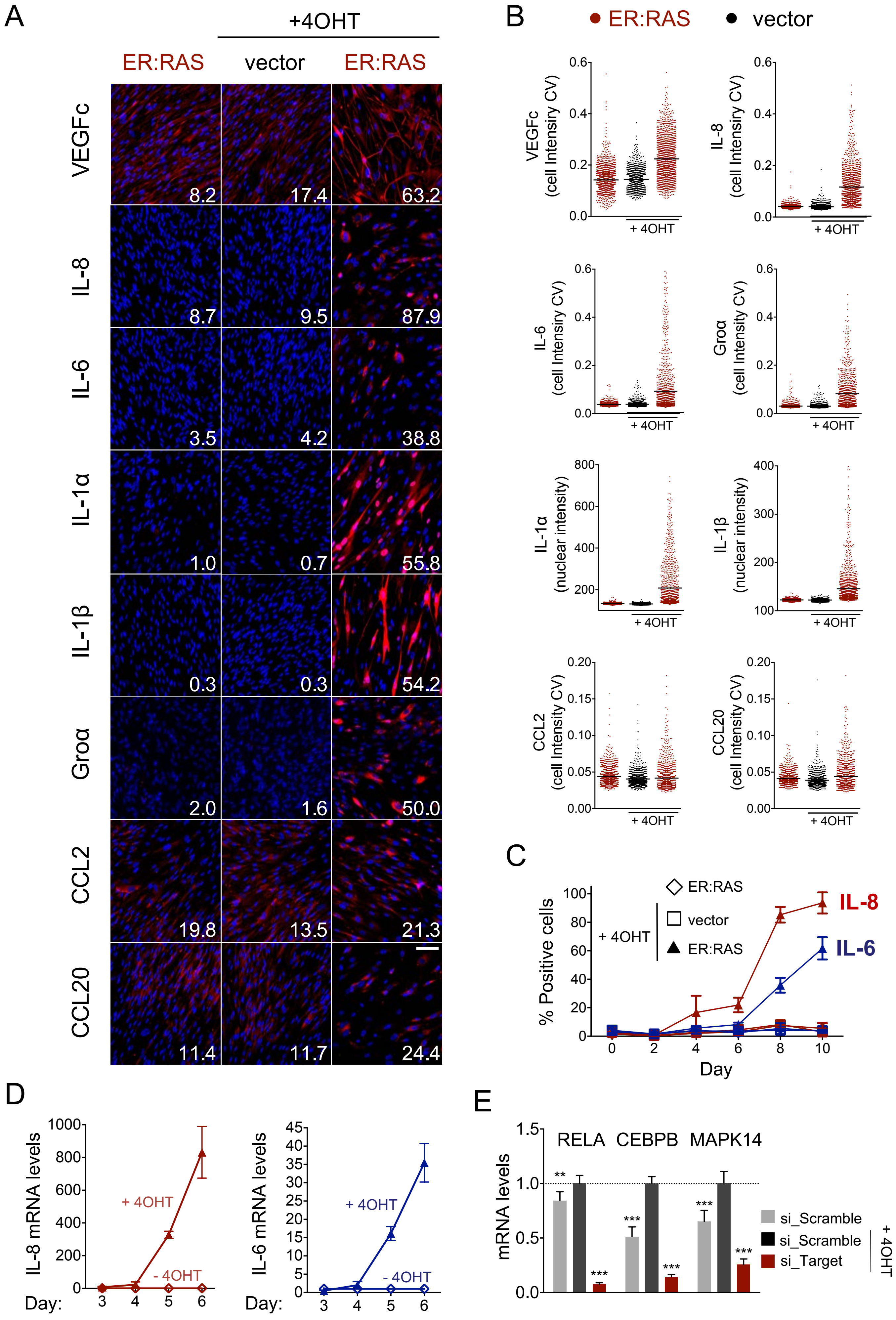

A
+4OHT
ER:RAS
vector
ER:RAS
VEGFc
8.2
17.4
63.2
IL-8
8.7
9.5
87.9
IL-6
3.5
4.2
38.8
IL-1α
1.0
0.7
55.8
IL-1β
0.3
0.3
54.2
Groα
2.0
1.6
50.0
CCL2
19.8
13.5
21.3
CCL20
11.4
11.7
24.4
B
ER:RAS
vector
VEGFc (cell Intensiry CV)
IL-8 (cell Intensiry CV)
IL-6 (cell Intensiry CV)
Groα (cell Intensiry CV)
IL-1α (nuclear intensity)
IL-1β (nuclear intensity)
CCL2 (cell Intensiry CV)
CCL20 (cell Intensiry CV)
+ 4OHT
C
ER:RAS
+ 4OHT
vector
ER:RAS
IL-8
IL-6
% Positive cells
Day
D
IL-8 mRNA levels
IL-6 mRNA levels
+ 4OHT
- 4OHT
Day:
E
RELA
CEBPB
MAPK14
mRNA levels
si_Scramble
si_Scramble
si_Target
+ 4OHT

Figure S1, related to Figure 1. IL-8 and IL-6 as readouts for a SASP screen.

(A) Representative IF images of the indicated SASP markers (red) co-stained with DAPI (blue) in IMR90 vector or IMR90 ER:RAS cells treated with 4OHT for 8 days. Percentage of cells positive for the indicated marker for each sample is given at the bottom right of each image and is a mean of 3 replicate wells of a single experiment. Scale bar: 100 µm. (B) Distribution of single cell intensities of each marker within each condition from (A) in a single sample-well of a 96-well plate. (C) Time-course of IL-8 and IL-6 measured by quantitative IF in IMR90 vector or IMR90 ER:RAS cells. Day 0 = day of 4OHT induction. Values are mean±SD from 3 replicate wells of a single experiment. (D) Time-course of IL-8 and IL-6 measured by RT-qPCR in IMR90 ER:RAS cells. For each time-point the conditions were normalized to IMR90 ER:RAS not treated with 4OHT. Data represent mean±SD (n=3). (E) mRNA analysis by RT-qPCR of indicated known SASP regulators 5 days after 4OHT induction of IMR90 ER:RAS cells transfected with siRNAs targeting the indicated genes, normalized and compared to si_Scramble + 4OHT. Data represent mean±SD (n=4); $^{**}p < 0.01$, $^{***}p < 0.001$. Two-way ANOVA (Dunnett's test).

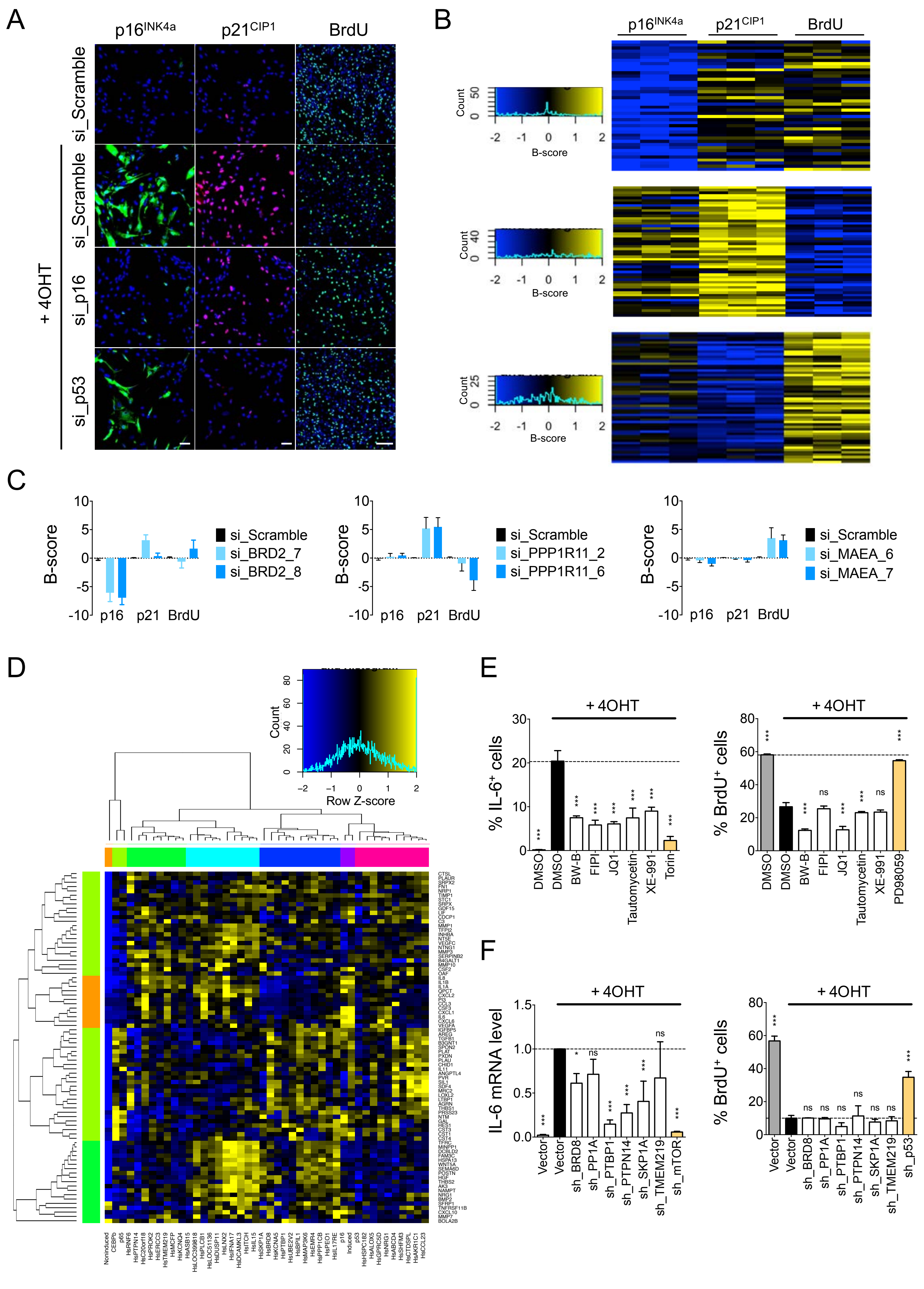
A
p16INK4a
p21CIP1
BrdU
si_Scramble
+ 4OHT
si_Scramble
si_p16
si_p53
B
p16INK4a
p21CIP1
BrdU
Count
B-score
C
B-score
p16
p21
BrdU
si_Scramble
si_BRD2_7
si_BRD2_8
si_PPP1R11_2
si_PPP1R11_6
si_MAEA_6
si_MAEA_7
D
Count
Row Z-score
E
+ 4OHT
% IL-6+ cells
DMSO
BW-B
FIPI
JQ1
Tautomycetin
XE-991
Torin
% BrdU+ cells
PD98059
F
+ 4OHT
IL-6 mRNA level
Vector
sh_BRD8
sh_PP1A
sh_PTBP1
sh_PTPN14
sh_SKP1A
sh_TMEM219
sh_mTOR
% BrdU+ cells
sh_p53

Figure S2, related to Figure 2. A subset of the screen candidate genes regulates the SASP but not the senescence arrest.

(A) Representative IF images of the indicated senescence markers following knockdown of known senescence regulators. Scale bar: 50 µm ($p16^{INK4A}$ and $p21^{CIP1}$) and 200 µm (BrdU). (B) *K*-means clustering of the SASP-repressing siRNAs. Heatmap of cluster 2 (top), 3 (middle) and 4 (bottom) showing B-score expression for each replicate experiment (column). Each row reflects the measures from one siRNA. (C) B-scores of 2 independent siRNAs targeting genes representative of cluster 2 (left), 3 (middle) and 4 (right). Mean +SD. (D) A detailed diagram of Figure 2D, left, showing 38 genes and additional controls clustering according to expression of the indicated SASP components. (E) IF analysis of IL-6 or BrdU incorporation 8 or 5 days after 4OHT induction, respectively, and treated with DMSO or 6 chemical compounds. Orange bars represent controls, either treatment with 25 nM Torin1 (to prevent IL-6 induction), or treatment with 20 µM PD98059 to prevent growth arrest (resulting in higher BrdU incorporation). Data represent mean±SD (n=3); ***$p < 0.001$; ns,not significant. Comparisons to DMSO + 4OHT. (F) Expression levels of IL-6 measured by RT-qPCR (left) and IF analysis of BrdU incorporation (right) 6 days after 4OHT induction of IMR90 ER:RAS cells that were stably infected with the indicated pGIPZ vectors. Orange bars represent controls, either shmTOR (that prevents IL-6 induction) or shp53 (that blunts growth arrest, resulting in higher BrdU incorporation). Data represent mean±SD (n=3); *$p < 0.05$, ***$p < 0.001$. Comparisons to Vector + 4OHT. One-way ANOVA (Bonferroni test) was used in (E) and (F) to calculate statistical significance.

Table S1, related to Figure 2. Genes present in cluster 1 with ≥ 2 siRNAs.

Genes
ABCD4
AKR1C1
ALOX5
ASB15
BPIL1
BRD4
BRD8
C20orf18
CBR3
CCL23
CTDSPL
CYP20A1
DCAMKL3
DCK
DUSP10
EMR4
ERCC3
GPR133
GPRC5D
HSPC182
IFNA17
IL15
IL17RE
ITCH
ITGB2
KCNA5
KCNQ4
LNX2
LOC399818
LOC51136
MAP3K6
MCFP
NRG1
PEO1
PHF6
PLCB1
PPP1CB
PROK2
PTBP1
PTPN14
RNF6
SACM1L
SERPINB2
SHFM3

SKP1A
TLR3
TMEM219
TRIM17
UBE2V2
UNG

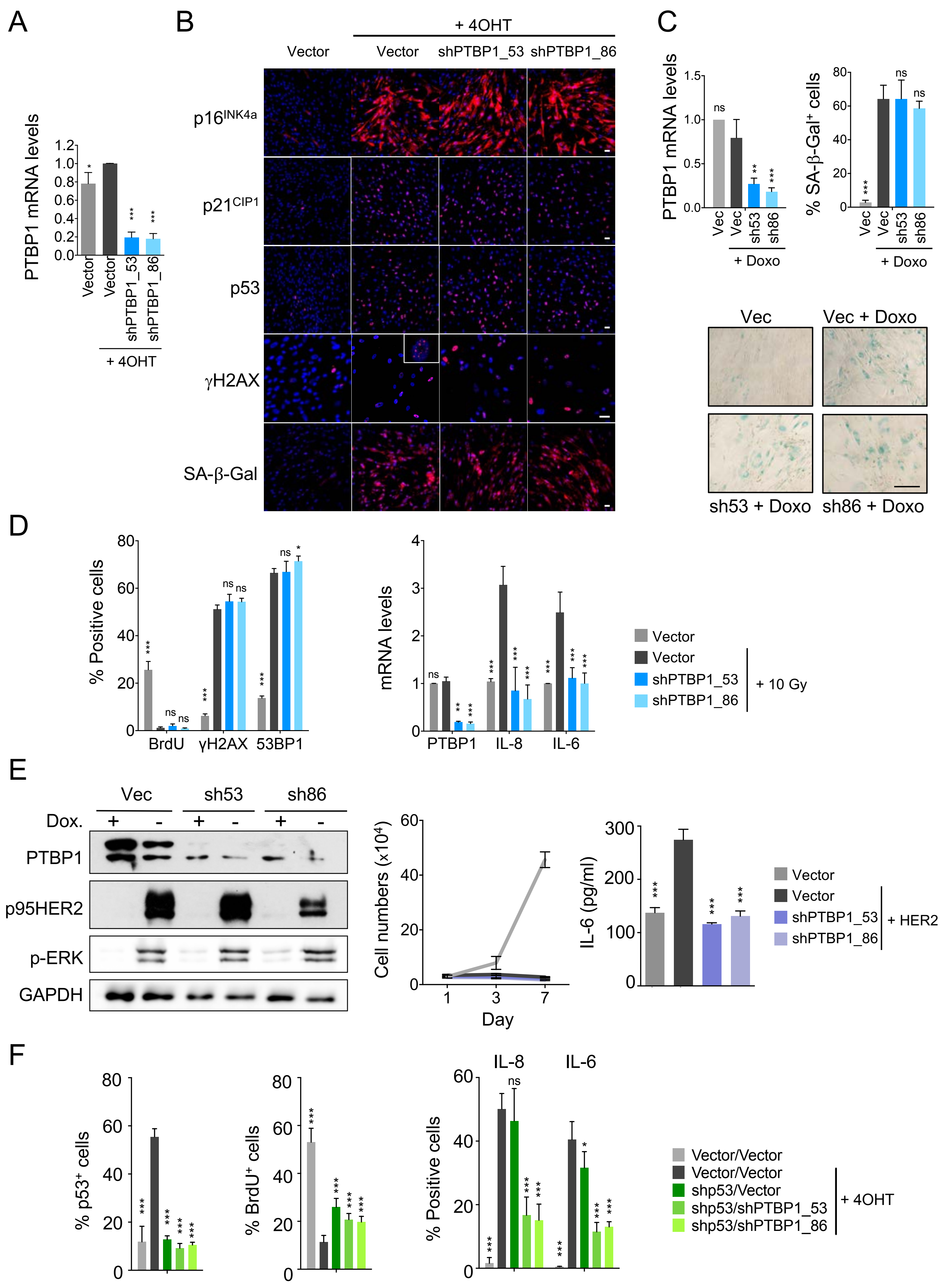

A
PTBP1 mRNA levels
Vector
Vector
shPTBP1_53
shPTBP1_86
+ 4OHT
B
+ 4OHT
Vector
Vector
shPTBP1_53
shPTBP1_86
p16INK4a
p21CIP1
p53
γH2AX
SA-β-Gal
C
PTBP1 mRNA levels
% SA-β-Gal+ cells
Vec
Vec
sh53
sh86
+ Doxo
Vec
Vec + Doxo
sh53 + Doxo
sh86 + Doxo
D
% Positive cells
BrdU
γH2AX
53BP1
mRNA levels
PTBP1
IL-8
IL-6
Vector
Vector
shPTBP1_53
shPTBP1_86
+ 10 Gy
E
Vec
sh53
sh86
Dox.
PTBP1
p95HER2
p-ERK
GAPDH
Cell numbers (x10⁴)
Day
IL-6 (pg/ml)
Vector
Vector
shPTBP1_53
shPTBP1_86
+ HER2
F
% p53+ cells
% BrdU+ cells
% Positive cells
IL-8
IL-6
Vector/Vector
Vector/Vector
shp53/Vector
shp53/shPTBP1_53
shp53/shPTBP1_86
+ 4OHT

Figure S3, related to Figure 3. PTBP1 regulates the SASP without affecting growth arrest.

(A) RT-qPCR analysis of PTBP1 expression 6 days after 4OHT induction of IMR90 ER:RAS cells infected with indicated pGIPz shRNA vectors, normalized and compared to Vector + 4OHT. Data represent mean±SD (n=3); *p < 0.05, ***p < 0.001. One-way ANOVA (Dunnett's test). (B) Representative IF images of the indicated senescence markers upon PTBP1 knockdown. Scale bar: 50 μm. (C) PTBP1 depletion in IMR90 cells undergoing doxorubicin-induced senescence. RT-qPCR analysis of PTBP1 expression (top left), normalized and compared to Vector + 4OHT. Quantification of SA-β-Galactosidase expression (top right) and representative images (bottom) 8 days after doxorubicin treatment. Data represent mean±SD (n=3); **p < 0.01, ***p < 0.001; ns, not significant,. One-way ANOVA (Dunnett's test). Scale bar: 100 μm. (D) HFFF2 cells were infected with indicated pGIPZ empty vector or PTBP1 shRNAs and irradiated to induce senescence. Left: IF analysis of BrdU incorporation and DDR markers 5 days after irradiation. Right: RT-qPCR analysis of the indicated genes 9 days after irradiation, normalized to Vector. Data represent mean±SD (n=3); *p < 0.05, **p < 0.01, ***p < 0.001; ns, not significant. Comparisons to Vector + 10 Gy. Two-way ANOVA (Dunnett's test). (E) MCF7 Tet-Off/p95HER2 cells were infected with indicated pGIPZ empty vector or PTBP1 shRNAs and treated with doxycycline to prevent p95HER2-induced senescence. Left: Immunoblot of protein extracts to monitor PTBP1 knockdown, p95HER2 expression and ERK signaling activation 7 days after doxycycline removal. Middle: Growth arrest assessed by cell numbers at indicated days after doxycycline removal. Data represent mean±SD (n=3). Right: IL-6 levels assessed by ELISA of media conditioned for 7 days after doxycycline removal. Data represent mean±SD (n=3); ***p < 0.001. Comparisons to Vector + HER2 (+ doxycycline). One-way ANOVA (Bonferroni test). (F) IMR90 ER:RAS cells were stably infected with the indicated vectors 1:1 to monitor PTBP1 knockdown in shp53-mediated bypass of senescence growth arrest. Left and middle: IF analysis of p53 expression and BrdU incorporation 6 days after 4OHT treatment. Right: IF analysis of SASP 8 days after 4OHT treatment. Data represent mean±SD (n=3); *p < 0.05, ***p < 0.001; ns, not significant. Comparison to Vector/Vector + 4OHT. One-way ANOVA (Dunnett's test).

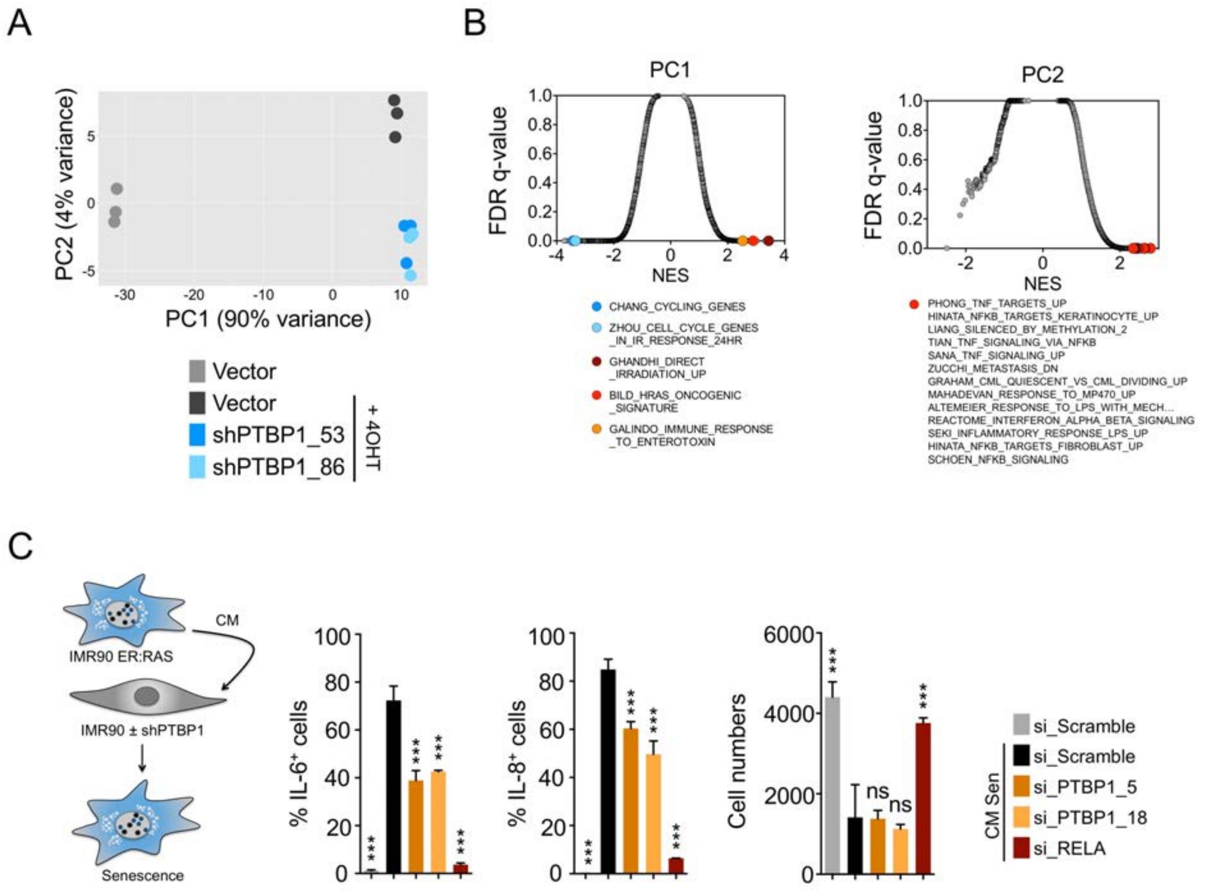

Figure S4, related to Figure 4. PTBP1 knockdown inhibits SASP-mediated effects.

(A – B) Global transcriptional profiling of PTBP1 depleted cells. PCA of the expression of the top 1,000 genes in proliferating cells, senescent cells and senescent cells lacking PTBP1. Each dot represents one of 3 independent replicates (A). The PCA axis separating the 4OHT treated samples from the proliferating samples (PC1) is significantly enriched in signatures associated with senescence and significantly depleted of signatures associated with proliferation. The PCA axis separating vector from shPTBP1 samples (PC2) is significantly depleted of signatures associated with inflammatory factors (represented by red dots). (C) SASP and growth arrest in paracrine senescence following PTBP1 depletion. Experimental design (left). IMR90 cells were transfected with indicated siRNAs and after 2 days treated with media conditioned by the specified senescent states. The SASP was assessed by quantitative IF and the growth arrest by cell numbers. Data represent mean±SD (n=3); ***$p < 0.001$; ns, not significant. Comparisons to si_Scramble + senescent cell CM. One-way ANOVA (Dunnett's test).

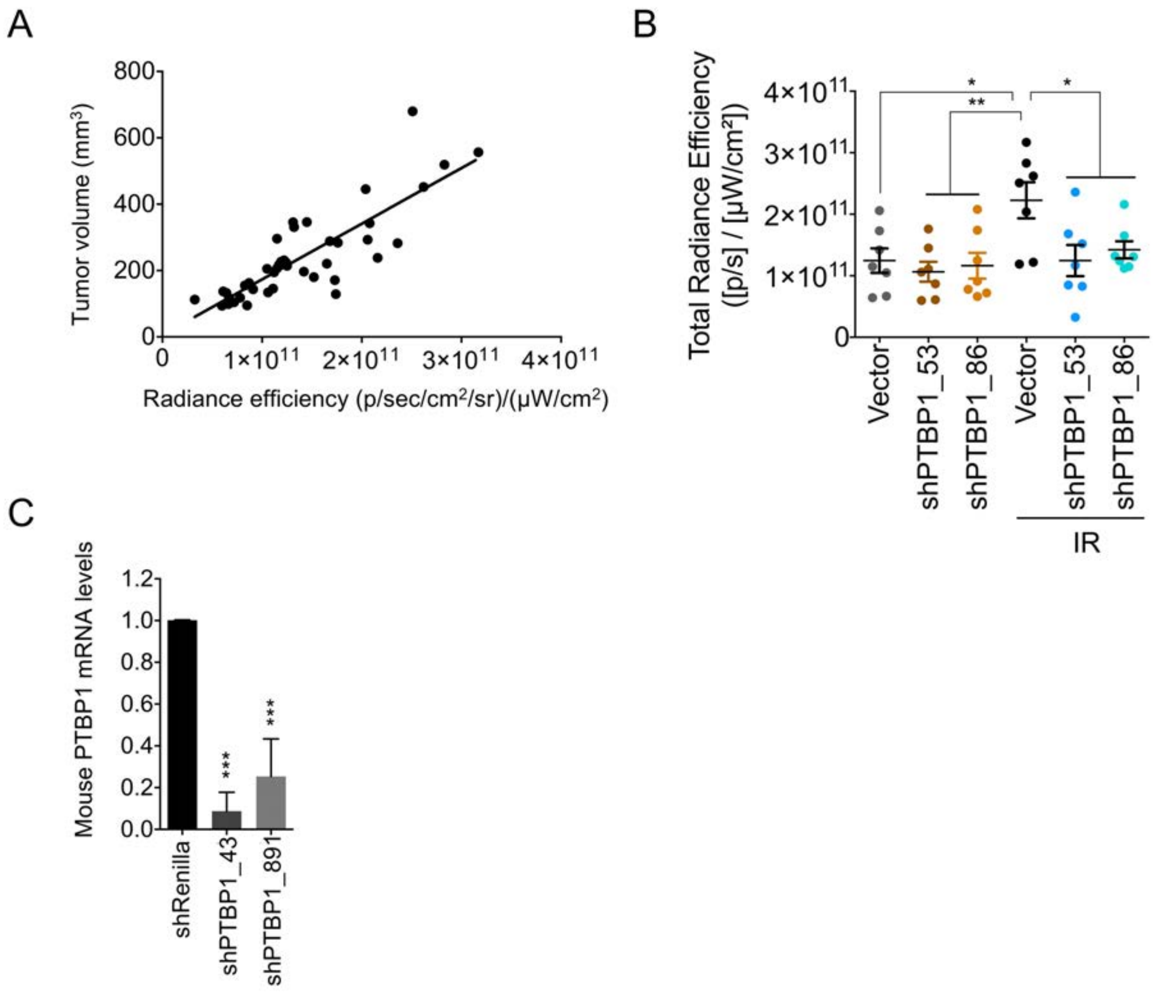

Figure S5, related to Figure 5. PTBP1 knockdown inhibits SASP-mediated tumor promotion.

(A) Comparison of tumor volume measurements using caliper (y-axis) and IVIS readings (x-axis). Linear regression: $R^2 = 0.6913$, $p < 0.0001$. Each graph symbol corresponds to the measurement of a single mouse (n=42 mice total). (B) Tumor growth measured by radiance efficiency at endpoint. Each graph symbol corresponds to the measurement of a single mouse (n=7 per group). *p < 0.05, **p < 0.01. Comparisons to Vector/IR. One-way ANOVA (Dunnett's test). (C) RT-qPCR analysis of mouse PTBP1 in MEFs (Mouse Embryonic Fibroblasts) stably infected with the two distinct PTBP1-targeting shRNAs. Data represent mean±SD (n=3); ***p < 0.001. Comparison to shRenilla. One-way ANOVA (Dunnett's test).

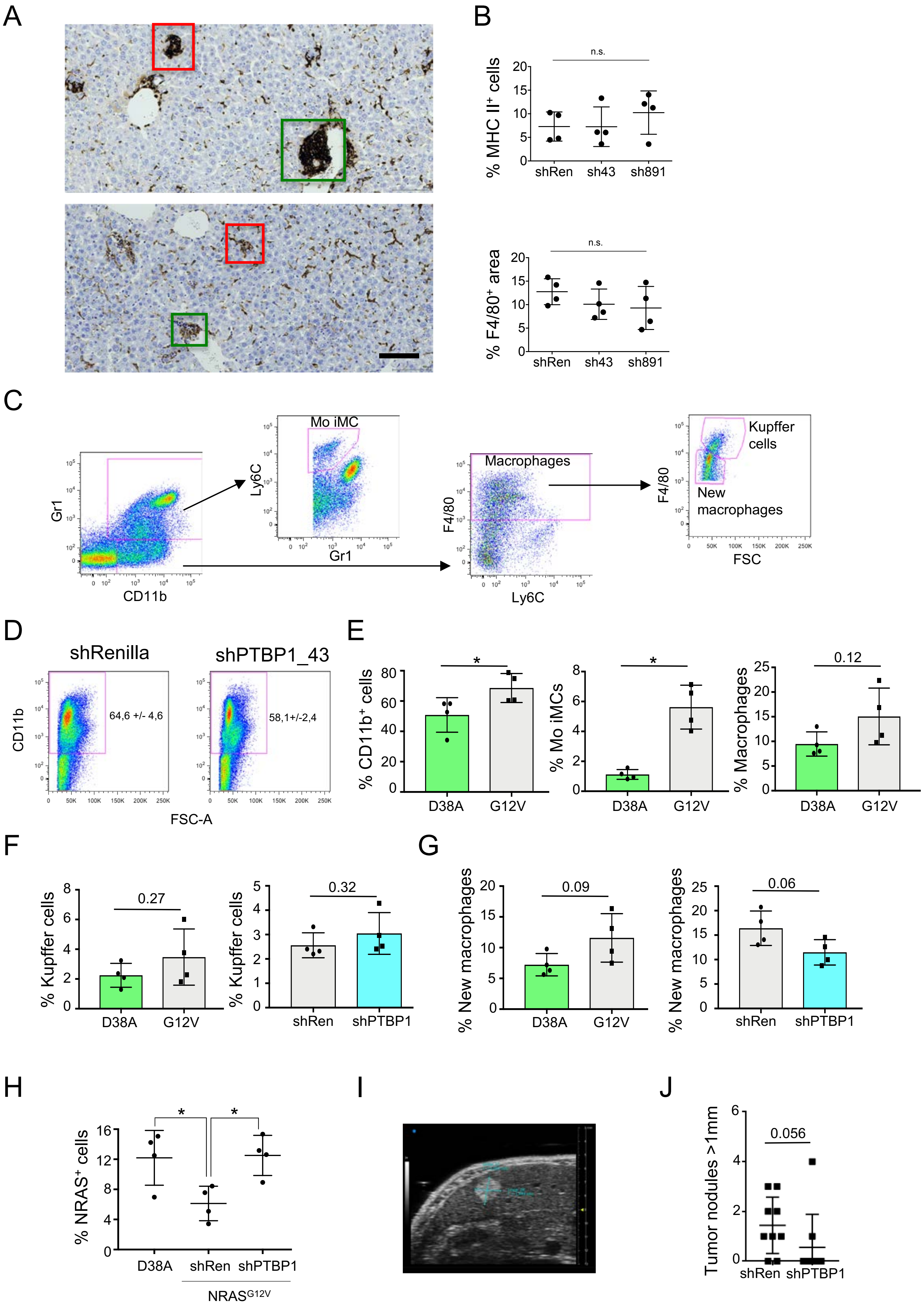
A
B
n.s.
% MHC II+ cells
shRen
sh43
sh891
% F4/80+ area
C
Gr1
CD11b
Mo iMC
Ly6C
Macrophages
F4/80
Kupffer cells
New macrophages
FSC
D
shRenilla
shPTBP1_43
64,6 +/- 4,6
58,1+/-2,4
FSC-A
E
% CD11b+ cells
% Mo iMCs
% Macrophages
D38A
G12V
0.12
F
% Kupffer cells
0.27
0.32
shRen
shPTBP1
G
% New macrophages
0.09
0.06
H
% NRAS+ cells
D38A
shRen
shPTBP1
NRASG12V
I
J
Tumor nodules >1mm
0.056

Figure S6, related to Figure 6. PTBP1 knockdown affects immune infiltration without increasing tumorigenesis.

(A – B) Analysis of liver infiltrating immune cells by IHC. IHC stained for MHC II and F4/80 in liver sections of shRenilla control with characteristic myeloid aggregate formation. The green box exemplifies big plaques (MHC II >70 µm; F4/80 >40µm) and the red box exemplifies small plaques (MHC II 40 - 70 µm; F4/80 30 - 40 µm). Scale Bar: 100 µm (A). Relative densitometric measurement of whole MHC II and F4/80 IHC stained liver sections. Here the total positive area is measured as opposed to number of size-separated aggregates given in Figure 4C. Data represent mean±SD (n=4 mice per condition); ns, not significant. Comparisons to $NRAS^{G12V}$_shRenilla (B). (C – G) Analysis of liver infiltrating immune cells by flow cytometry. Representative dot plots and gating strategy of monocytic iMC, macrophages, Kupffer cells and infiltrating new macrophages. Macrophages can be distinguished in liver resident Kupffer cells (F4/80high) and infiltrating new macrophages ($F4/80^{+}$) (C). Representative dot plots of infiltrating $CD11b^{+}$ myeloid cells in Ras^{G12V}_shRen and Ras^{G12V}_shPTBP1.43 (D). Quantification of indicated infiltrating cells (E). Quantification of Kupffer cells (F). Quantification of new macrophages (G). (E – G) Data represent mean±SD (n=4 mice per condition); *p < 0.05. (H) Quantification of $NRAS^{+}$ cells in $NRAS^{G12V,\ D38A}$ (n=4), $NRAS^{G12V}$_shRen (n=4) and $NRAS^{G12V}$_shPTBP1 (n=4) to monitor clearance of senescent cells upon PTBP1 knockdown. Data represent mean±SD; *p < 0.05. (I – J) Long-term tumor development in mice upon injection with $NRAS^{G12V}$_shRen (n=10) and $NRAS^{G12V}$_shPTBP1 (n=9). Example of detected tumour with sonography in mice (I). Quantification of tumor nodules at endpoint. Data represent mean±SD (J).

One-way ANOVA (Bonferroni test) was used in (B), (E – H) and (J) to calculate statistical significance.

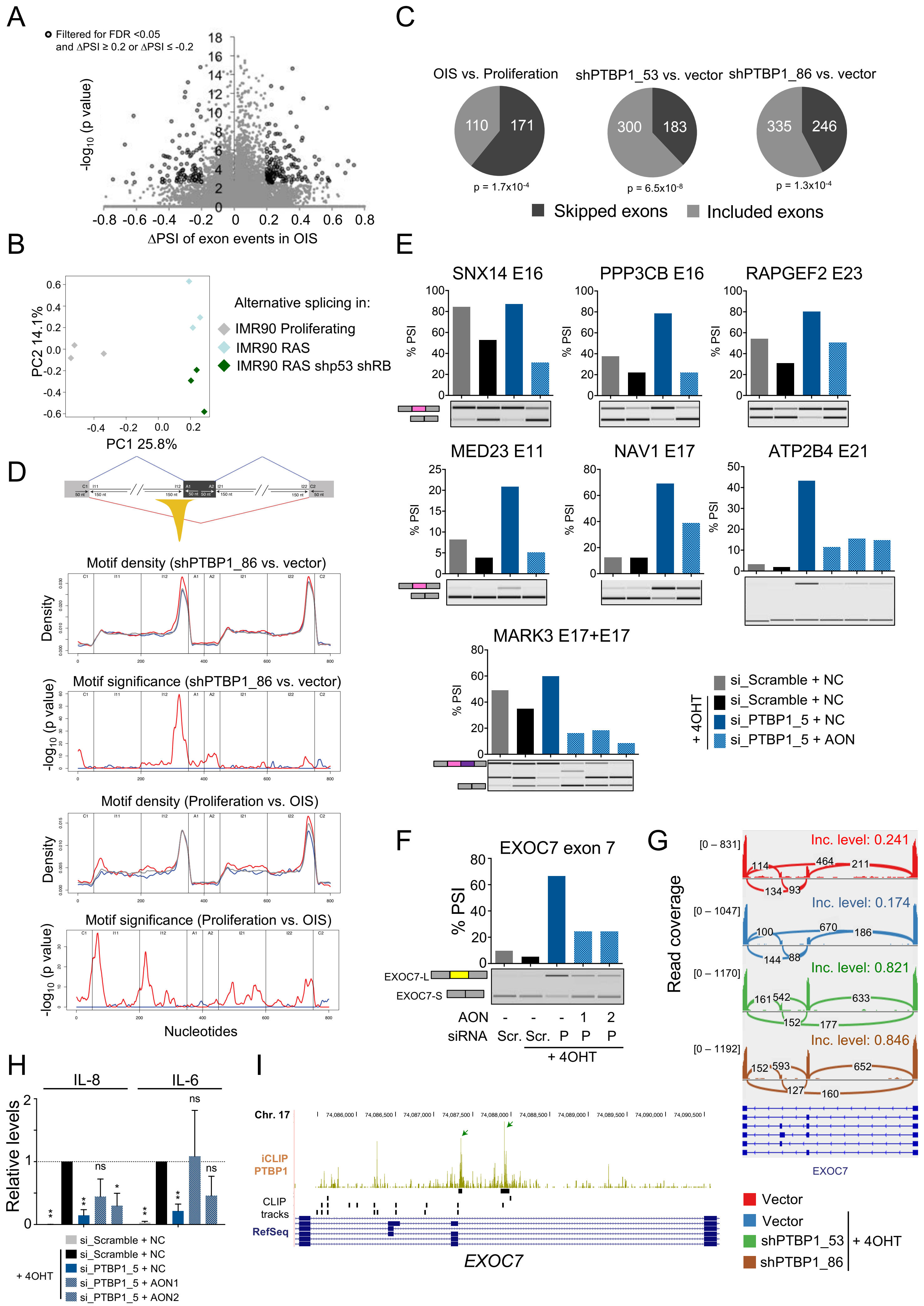

A
Filtered for FDR <0.05 and ΔPSI ≥ 0.2 or ΔPSI ≤ -0.2
-log10 (p value)
ΔPSI of exon events in OIS
B
PC2 14.1%
PC1 25.8%
Alternative splicing in:
IMR90 Proliferating
IMR90 RAS
IMR90 RAS shp53 shRB
C
OIS vs. Proliferation
shPTBP1_53 vs. vector
shPTBP1_86 vs. vector
110
171
300
183
335
246
p = 1.7x10-4
p = 6.5x10-8
p = 1.3x10-4
Skipped exons
Included exons
D
Motif density (shPTBP1_86 vs. vector)
Motif significance (shPTBP1_86 vs. vector)
Motif density (Proliferation vs. OIS)
Motif significance (Proliferation vs. OIS)
Density
-log10 (p value)
Nucleotides
E
SNX14 E16
PPP3CB E16
RAPGEF2 E23
MED23 E11
NAV1 E17
ATP2B4 E21
MARK3 E17+E17
% PSI
si_Scramble + NC
si_Scramble + NC
si_PTBP1_5 + NC
si_PTBP1_5 + AON
+ 4OHT
F
EXOC7 exon 7
% PSI
EXOC7-L
EXOC7-S
AON
siRNA
Scr.
Scr.
P
P
P
+ 4OHT
G
Read coverage
[0 – 831]
[0 – 1047]
[0 – 1170]
[0 – 1192]
Inc. level: 0.241
Inc. level: 0.174
Inc. level: 0.821
Inc. level: 0.846
EXOC7
Vector
Vector
shPTBP1_53
shPTBP1_86
+ 4OHT
H
IL-8
IL-6
Relative levels
ns
si_Scramble + NC
si_Scramble + NC
si_PTBP1_5 + NC
si_PTBP1_5 + AON1
si_PTBP1_5 + AON2
+ 4OHT
I
Chr. 17
iCLIP PTBP1
CLIP tracks
RefSeq
EXOC7

Figure S7, related to Figure 7. Regulation of alternative splicing by PTBP1 controls the SASP.

(A) Distribution of exon skipping events detected by RMATS and cut-offs used when comparing senescent to proliferating cells. (B) PCA of exon inclusion levels of datasets described in Tasdemir et al. (2016) to examine relative contribution of Ras activation and senescence. (C) Relative numbers of more skipped and more included amongst the significantly differentially spliced exons. (D) 1st panel: Scheme as in Figure 7B, top. 2nd and 4th panel: average (31bp moving window) PTBP1 RNA-binding motif density (# motifs per alternative splicing event) for exons with PSIs increasing upon PTBP1 knockdown (putatively repressed, red), exons with PSIs decreasing upon PTBP1 knockdown (putatively enhanced, blue), and exons with PSIs not altered upon PTBP1 knockdown (putatively not regulated, grey). 3rd and 5th panel**:** statistical significance for local PTBP1 RNA-binding motif enrichment in putatively repressed (red line) and putatively enhanced (blue line) exons. (E) Effect of AONs targeting the indicated exons on the splice-switch induced by PTBP1 knockdown. (F) Effect of AONs targeting EXOC7 exon 7 on the splice-switch induced by PTBP1 knockdown. (E and F) For mRNA analysis, IMR90 ER:RAS cells were transfected with AONs 2 days after 4OHT induction and RT-qPCR was performed 4 days after 4OHT induction. (G) Sashimi plot showing EXOC7 exon 7 splicing. RNA-seq data as in Figure 4A. Values are mean of 3 replicates. Blue boxes represent exons, empty blue box represents exon 7, and dotted lines represent introns (genome assembly hg19). (H) Timeline as in Figure 7F. Media were collected 6 days after 4OHT induction and SASP was evaluated by ELISA. Data represent mean±SD (n=3, independent generation of CM); $^{**}p < 0.01$; ns, not significant. Comparisons to NC, si_Scramble + NC+ 4OHT. Two-way ANOVA (Dunnett's test). (I) UCSC Genome Browser tracks visualizing public PTBP1 CLIP-seq data, around EXOC7 exon 7 (genome assembly hg19). Green arrows: PTBP1 binding sites potentially regulating exon 7 splicing. CLIP tracks, from top to bottom: HITS-CLIP PTBP1 HeLa; PAR-iCLIP PTBP1 293T; iCLIP PTBP1 293T dox-induc nSR100 KD; iCLIP PTBP1 293T dox-induc nSR100 OE.

Table S3, related to Figure 7. Alternatively spliced transcripts selected for siRNA SASP screen.

Alternatively spliced transcripts
ABLIM3
ANKRD27
APBA1
APH1A
ARVCF
ASAP2
ATP11C
ATP2B4
BTN3A2
C19orf12
CACNB1
COPZ2
CROCCP2
CTC-205M6.2
CTNND1
DCLK2
DMD
DPP4
DZIP1
ENAH
EP400
EPB41L3
EVC
EXOC1
EXOC7
FAM190B
FAM208B
FAM21B
FBXO25
FERMT2
FLOT1
FMNL2
GABBR1
GAPVD1
GNB5
HPS4
ITGA6
KIAA1217
KIAA1715
KIF3A
KLC1
KRIT1

LOXL1-AS1
LRRC27
LRRFIP2
MAGI2-AS3
MARK3
MCTP1
MED23
MEG3
MICAL2
MYL12A
MYO1B
NAV1
OGDH
PARP6
PARPBP
PBX1
PDLIM5
PICALM
PITPNM2
PPIP5K2
PPP1R12B
PPP3CB
PRKD1
PTBP2
PXN
R3HDM1
RAPGEF2
RAPH1
RNF38
ROBO3
RPL26
RPS24
RTN4
SEC31A
SLMO2
SMAP1
SMTN
SNX14
SSFA2
STAG3L3
STX3
SUCO
SVIL
TAPBP
TBC1D24
TNIK
TPM1
TRERF1

TTC7A
USP16
VPS13D
YIPF1
ZNF484

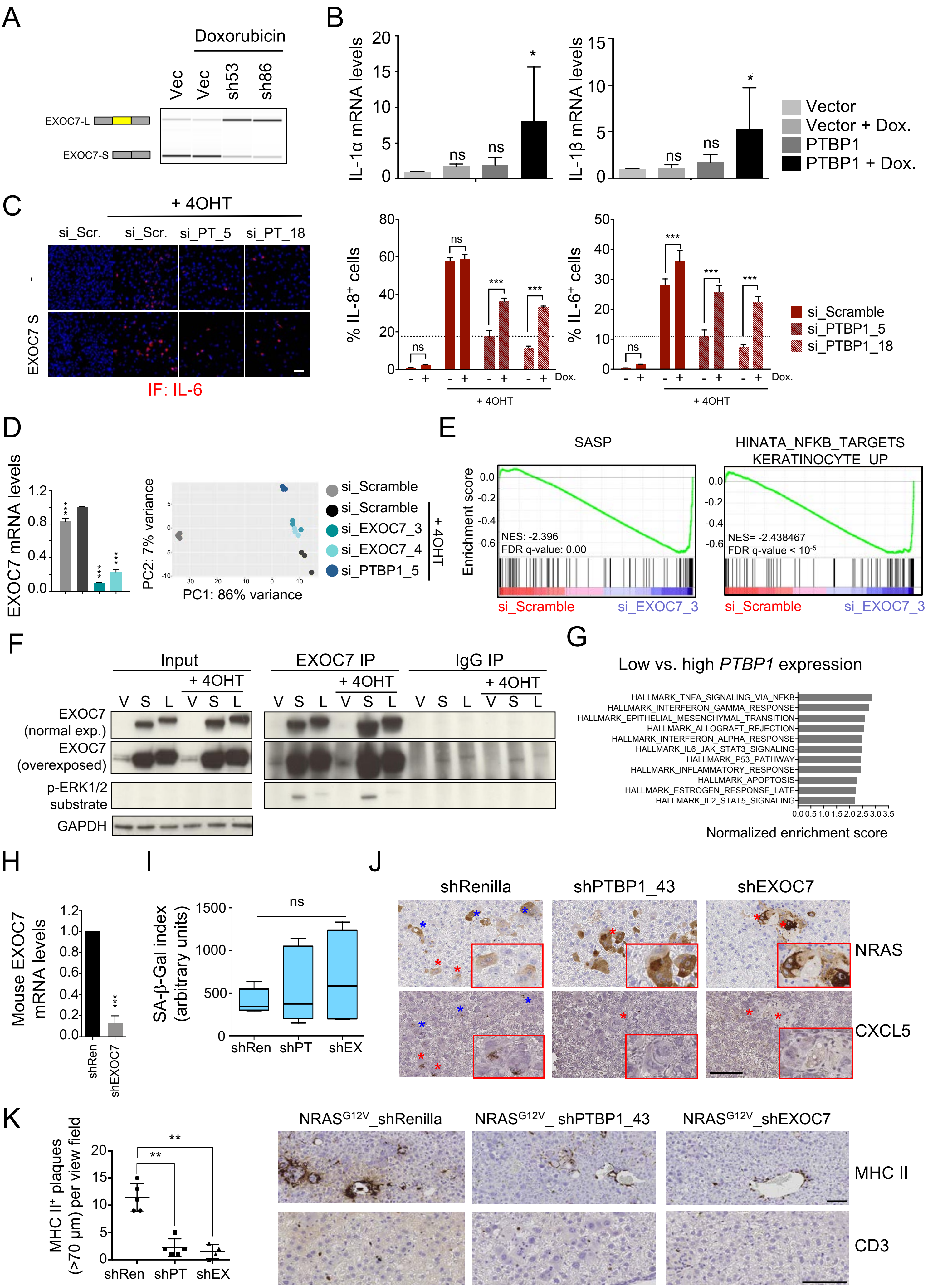
A
Doxorubicin
Vec
Vec
sh53
sh86
EXOC7-L
EXOC7-S
B
IL-1α mRNA levels
IL-1β mRNA levels
ns
*
Vector
Vector + Dox.
PTBP1
PTBP1 + Dox.
C
+ 4OHT
si_Scr.
si_Scr.
si_PT_5
si_PT_18
EXOC7 S
IF: IL-6
% IL-8+ cells
% IL-6+ cells
Dox.
+ 4OHT
si_Scramble
si_PTBP1_5
si_PTBP1_18
D
EXOC7 mRNA levels
PC2: 7% variance
PC1: 86% variance
si_Scramble
si_Scramble
si_EXOC7_3
si_EXOC7_4
si_PTBP1_5
+ 4OHT
E
SASP
HINATA_NFKB_TARGETS KERATINOCYTE_UP
Enrichment score
NES: -2.396
FDR q-value: 0.00
NES= -2.438467
FDR q-value < 10-5
si_Scramble
si_EXOC7_3
F
Input
EXOC7 IP
IgG IP
+ 4OHT
V S L V S L
EXOC7 (normal exp.)
EXOC7 (overexposed)
p-ERK1/2 substrate
GAPDH
G
Low vs. high PTBP1 expression
HALLMARK_TNFA_SIGNALING_VIA_NFKB
HALLMARK_INTERFERON_GAMMA_RESPONSE
HALLMARK_EPITHELIAL_MESENCHYMAL_TRANSITION
HALLMARK_ALLOGRAFT_REJECTION
HALLMARK_INTERFERON_ALPHA_RESPONSE
HALLMARK_IL6_JAK_STAT3_SIGNALING
HALLMARK_P53_PATHWAY
HALLMARK_INFLAMMATORY_RESPONSE
HALLMARK_APOPTOSIS
HALLMARK_ESTROGEN_RESPONSE_LATE
HALLMARK_IL2_STAT5_SIGNALING
Normalized enrichment score
H
Mouse EXOC7 mRNA levels
shRen
shEXOC7
I
SA-β-Gal index (arbitrary units)
ns
shRen
shPT
shEX
J
shRenilla
shPTBP1_43
shEXOC7
NRAS
CXCL5
K
MHC II+ plaques (>70 μm) per view field
shRen
shPT
shEX
NRASG12V_shRenilla
NRASG12V_ shPTBP1_43
NRASG12V_shEXOC7
MHC II
CD3

Figure S8, related to Figure 8. PTBP1 regulates alternative splicing of EXOC7 to control the SASP.

(A) EXOC7 splicing regulation by PTBP1 in other types of senescence. Doxorubicin-induced senescence is shown as an example. Alternative splicing analysis by RT-qPCR 8 days after doxorubicin treatment of IMR90 WT cells infected with the indicated pGIPz-based vectors. (B) SASP expression following PTBP1 overexpression in WT IMR90 cells. mRNA analysis by RT-qPCR 2 days after induction of PTBP1 expression with doxycycline (Dox). Data represent mean±SD (n=5); *p < 0.05; ns, not significant. Comparisons to Vector. One-way ANOVA (Dunnett's test). (C) Effect of EXOC7-S on the SASP downregulation caused by PTBP1 knockdown. Left: Representative IF images of IL-6 without doxycycline treatment (-) and with (EXOC7 S). Scale Bar: 100 µm. Right: Quantification of IF analysis. Data represent mean±SD (n=3); ***p < 0.001; ns, not significant. Two-way ANOVA (Bonferroni test). (D – E) SASP expression upon EXOC7 depletion. Left: mRNA analysis of EXOC7 by RT-qPCR. Data represent mean±SD (n=3); ***p < 0.001; ns, not significant. Comparisons to si_Scramble + 4OHT. One-way ANOVA (Dunnett's test). Right: PCA of the expression of the top 500 most variable genes in proliferating cells, senescent cells and senescent cells lacking EXOC7 or PTBP1. Each dot represents one of 3 independent replicates (D). NF-κB (left) and SASP (right) GSEA signatures in EXOC7 depleted cells + 4OHT compared to scramble transfected cells + 4OHT (E). (F) Comparison of EXOC7-S and EXOC7-L phosphorylation assessed by EXOC7 immunoprecipitation followed by immunoblotting as in Figure 8F. Here, including IP using IgG of the same species as EXOC7 antibody, serving as negative control for pulldown of EXOC7. (G) Top 11 hallmarks with NES > 2 and FDR < 0.05 in genes with expression positively correlating with PTBP1 expression in GTEx samples. (H) RT-qPCR analysis of mouse EXOC7 in MEFs stably infected an EXOC7-targeting shRNA. Data represent mean±SD (n=3); ***p < 0.001. One-way ANOVA (Dunnett's test). (I – K) Experimental design in Figure 8J. Quantification of SA-β-Galactosidase expression. Plots show median (line), upper and lower quartiles (boxes), and lines extending to highest and lowest observation (whiskers). ns, not significant. Comparisons to $NRAS^{G12V}$_shRenilla. One-way ANOVA (Dunnett's test) (I). Representative images of NRAS (top) and CXCL5 (bottom) positive cells on serial liver sections. NRAS measured by IHC and CXCL5 by *in situ* hybridisation. Asterisks denote $NRAS^{+}$ cells and the red insets zoom in the regions corresponding to red asterisks. Scale bar: 100 µm (J). Effect of EXOC7

knockdown on immune infiltration. Quantification of infiltrated MHC II^{+} cells forming large plaques (left) and representative images of IHC for MHC II and CD3 on liver sections. Data represent mean±SD; **$p < 0.01$. Comparisons to $NRAS^{G12V}$_shRenilla. One-way ANOVA (Bonferroni test). Scale bar: 100 µm (K).

Table S4, related to STAR METHODS. AON sequences used in this study.

Gene	Target Exon	AON Sequence
MARK3	MARK3 exon 16	AAUCCUACCUUUUGAUAAACCUGAAUGACAUG
MARK3	MARK3 exon 17	AUAUUCAGUUGGAAGCCUCAAUGAACAGAAUGUA
MARK3	MARK3 exon 17	GUUCCUCUCAUAUUCAGUUGGAAGC
NAV1	NAV1 exon 17	AUUGCAUCCUCUCCUCAGCCUGAAGAAG
PPP3CB	PPP3CB exon 16	UUUUCAGCCUCAAUAGCCUCAACUGUG
RAPGEF2	RAPGEF2 exon 23	CAAACUCCGCCAUUUCUUCUUCCUGUAA
RNF38	RNF38 exon 4	GUAGAGAUGCUGAAUUGGCCCCGG
RNF38	RNF38 exon 4	GAUGGCCAGGUAGAGAUGCUGAAUU
ATP2B4	ATP2B4 exon 21	UAAAAGAGGCUCCCGUCUGGAAUGUGUUA
ATP2B4	ATP2B4 exon 21	GAAGAUUUGACUGGUGCAACUGCUACAUA
ATP2B4	ATP2B4 exon 21	AUGUUCUGUCGCCUUAGGACUCCCUUAAAAG
EXOC7	EXOC7 exon 7	UACUGUUUCAGAAGGUUCUGAGCCUUA C
EXOC7	EXOC7 exon 7	UCUAGACCAUGCUGGGAAUACUGUU
MED23	MED23 exon 11	CUGAGCAAUGUUCAAGGUCUAGAGCAU
R3HDM1	R3HDM1 exon 18	AGACGACAGGUGCGCCAUGAGAAG
R3HDM1	R3HDM1 exon 18	GGAUGUGAGAAAGAGAGCCUGUAGAUGAC
SNX14	SNX14 exon 16	UUAGGCUACCUCUGCAAUAACAGGUAUGU
STX3	STX3 exon 10	UUAAUUCAGCCCAACGGAAAGUCCAAU
STX3	STX3 exon 10	UCAAUGCUAAAAUGCCCAGCAACAC
N/A	NC	CCUUCCCUGAAGGUUCCUCC

Table S5, related to STAR METHODS. RT-qPCR primer sequences.

Target	Forward primer	Reverse primer	Use
BRD8	TTGGGTATCAGTTAGCAGAGCA	TCAACAGTTTCCACCACTTCTC	Total
CCL20	GGCGAATCAGAAGCAGCAAGCAAC	ATTGGCCAGCTGCCGTGTGAA	Total
EXOC7	GGAGACGCTGTCGTTTATCCG	TGCAGCCTCTGTAGGTTCTCT	Total
IL1A	AGTGCTGCTGAAGGAGATGCCTGA	CCCCTGCCAAGCACACCCAGTA	Total
IL1B	TGCACGCTCCGGGACTCACA	CATGGAGAACACCACTTGTTGCTCC	Total
IL6	CCAGGAGCCCAGCTATGAAC	CCCAGGGAGAAGGCAACTG	Total
IL8	GAGTGGACCACACTGCGCCA	TCCACAACCCTCTGCACCCAGT	Total
INHBA	CGGCGCTTCTGAACGCGATC	GCTGTTCCTGACTCGGCAAACGT	Total
MMP1	TGTGGTGTCTCACAGCTTCC	CGCTTTTCAACTTGCCTCCC	Total
MMP3	TGTTCGTTTTCTCCTGCCTGT	CAGCAGCCCATTTGAATGCC	Total
PP1A	ACTACGACCTTCTGCGACTAT	AGTTCTCGGGGTACTTGATCTT	Total
PTBP1	AGCGCGTGAAGATCCTGTTC	CAGGGGTGAGTTGCCGTAG	Total
PTPN14	GGACAAATTCGCTAATGAGCCT	TAATCGCCCTTCAAGCACATC	Total
RPS14	CTGCGAGTGCTGTCAGAGG	TCACCGCCCTACACATCAAACT	Total
SKP1A	GACCATGTTGGAAGATTTGGGA	TGCACCACTGAATGACCTTTT	Total
TMEM219	ACGGTCCAGACAGGAACAAGA	CTGAGCAGGATATGGGTGGC	Total
UBE2V2	AGTTCCTCGTAATTTTCGCTTGT	CCCCAGCTAACTGTACCGT	Total
VEGFC	AGAGAACAGGCCAACCTCAA	TGGCATGCATTGAGTCTTTC	Total
ATP2B4	GATCCTCTGGTTCCGGGG	GCTGTGGATGGACTTTTGGT	AS
EXOC7	TACTCCCCTGCTATCCCCAA	ATGTAGGCATCGGTCTCCAC	AS
MARK3	CTGTCCCATGAAGCCACAC	TTTCATGCTCCAGGTGAAGC	AS
MED23	GTTGAGATATGTATTGGAGCAGC	TCAGATCGCTCCATGGCATA	AS
NAV1	CCACGGACGATGTTCACG	ATTCACCAGGCTCTGCTCAA	AS
PPP3CB	AGGAGAGTGAAAGTGTGCTGA	TGTACAGCATCTTTCCGAGGT	AS
RAPGEF2	ATGGCTTCAGTGAACATGGA	GCTTCACTTTTCGAGCCATTTG	AS
R3HDM1	TGGAAGCAGCAAAAGCATAGG	TGGTTGTTGAGTCATAGGAGGA	AS
SNX14	GCAGAATCACCAACACGCAA	GCAACATAGCTCCCTCCATTG	AS
STX3	GAAGGCACGAGATGAAACGA	CATCTGAAGACAAGGGTGGC	AS

Total, total mRNA
AS, alternative splicing
